# Removal of Aniline and Benzothiazole Wastewaters Using an Efficient MnO_2_/GAC Catalyst in a Photocatalytic Fluidised Bed Reactor

**DOI:** 10.3390/ma14185207

**Published:** 2021-09-10

**Authors:** Cristian Ferreiro, Natalia Villota, José Ignacio Lombraña, María J. Rivero, Verónica Zúñiga, José Miguel Rituerto

**Affiliations:** 1 Department of Chemical Engineering, Faculty of Science and Technology, University of the Basque Country UPV/EHU, Barrio Sarriena s/n, 48940 Leioa, Spain; ji.lombrana@ehu.eus; 2 Department of Chemical and Environmental Engineering, Faculty of Engineering Vitoria-Gasteiz, University of the Basque Country UPV/EHU, Nieves Cano 12, 01006 Vitoria-Gasteiz, Spain; natalia.villota@ehu.eus; 3 Department of Chemical and Biomolecular Engineering, University of Cantabria, 39005 Santander, Spain; riveromj@unican.es; 4 General Química, S.A.U. (Grupo Dynasol), 01213 Lantaron, Spain; veronica.zuniga@repsol.com (V.Z.); jrituertol@repsol.com (J.M.R.)

**Keywords:** MnO_2_/GAC composite, fluidised bed photoreactor, aniline, benzothiazole, sustainable photocatalysis, industrial wastewater

## Abstract

This work presents an efficient method for treating industrial wastewater containing aniline and benzothiazole, which are refractory to conventional treatments. A combination of heterogeneous photocatalysis operating in a fluidised bed reactor is studied in order to increase mass transfer and reduce reaction times. This process uses a manganese dioxide catalyst supported on granular activated carbon with environmentally friendly characteristics. The manganese dioxide composite is prepared by hydrothermal synthesis on carbon Hydrodarco^®^ 3000 with different active phase ratios. The support, the metal oxide, and the composite are characterised by performing Brunauer, Emmett, and Teller analysis, transmission electron microscopy, X-ray diffraction analysis, X-ray fluorescence analysis, UV–Vis spectroscopy by diffuse reflectance, and Fourier transform infrared spectroscopy in order to evaluate the influence of the metal oxide on the activated carbon. A composite of MnO_2_/GAC (3.78% in phase α-MnO_2_) is obtained, with a 9.4% increase in the specific surface of the initial GAC and a 12.79 nm crystal size. The effect of pH and catalyst load is studied. At a pH of 9.0 and a dose of 0.9 g L^−1^, a high degradation of aniline and benzothiazole is obtained, with an 81.63% TOC mineralisation in 64.8 min.

## 1. Introduction

One challenge associated with wastewater treatment is the presence of recalcitrant compounds, which are difficult to remove through conventional technologies [1]. Unfortunately, many of these pollutants play a key role in everyday-use products and are continuously added to aquatic environments through anthropogenic activities in sectors such as agriculture, industry, transport, and energy [2,3,4]. Recalcitrant contaminants broadly consist of high-molecular-weight hydrophobic molecules, including alcohols, phenols, and nitrogenous and sulphur compounds [5].

These emission sources without proper water treatments increase recalcitrant pollutants’ concentration in water, consequently damaging the environment and human health [6]. Therefore, degrading these refractory pollutants has become one of the challenges listed in the Sustainable Development Goals (SDGs), more specifically in SDG 6, towards mitigating their environmental impact by 2030. For this purpose, cost-effective treatment strategies should be developed to remove and mineralise these hazardous substances [7,8].

This study focuses on the removal of aniline (ANI) and benzothiazole (BTH) from industrial effluents. These two recalcitrant pollutants are commonly found in the leather and wood industry as well as in rubber chemicals production, specifically as raw materials for the synthesis of vulcanisation accelerators. In particular, these two pollutants have adverse effects on the health of organisms in aquatic environments and may also be tumour inducers and allergens [6,9]. In addition, BTH (included in Contaminant Candidate List 4) is not only found in industrial effluents but can also be detected in domestic wastewater and airport runoff water [10,11,12]. Bioremediation studies have reported uncertainty about the biodegradation of both compounds, especially aniline, owing to its genotoxic character. For this reason, conventional biological treatment systems or active sludge has failed to metabolise these pollutants [13,14].

Several technologies have been used to treat water containing ANI or BTH, such as coagulation [15], activated carbon adsorption [16,17,18], ozonation [9,19,20], photocatalysis [21,22,23], wet oxidation [24,25], and membrane separation [26]. However, the implementation of these technologies has some limitations, such as the adsorption capacity of the adsorbent, high disposal costs, high energy consumption, and low efficiency [27]. In membrane separation, zero-waste generation is unfeasible, because this process produces effluents with a high concentration of the target pollutants [26]. Moreover, adsorption processes only transfer the pollutant to a new phase (solid waste), which needs further treatment [28,29,30].

To overcome these major obstacles, photocatalytic degradation with new semiconductor materials has recently attracted worldwide attention as a green technology capable of degrading low-biodegradability organic and inorganic pollutants from industrial effluents [1,31,32]. Photocatalysis is a promising technology for degrading highly polluting compounds into other harmless by-products or even for mineralising them into CO_2_ and H_2_O. In addition, this low-cost technology can operate at room temperature [33,34,35].

Semiconductor photocatalytic materials are highly effective for treating wastewater containing recalcitrant pollutants when irradiated by ultraviolet (UV) or visible light. Some of the most widely used photocatalysts include TiO_2_, ZnO, and SnO [36,37,38]. In addition to the aforementioned catalysts, transition metals, such as copper, vanadium, nickel, and manganese, are being studied for their specific characteristics, including their affordable cost, effectiveness, abundance, and sustainability [39]. Catalysts such as TiO_2_ or ZnO, which could be widely used, are also associated with a series of drawbacks which affect the sustainability of their processes regarding the light source used to initiate the catalytic reaction. Their forbidden band (about 3.2 V) is so wide that they can only be activated under UV light, thus precluding visible light activation [40]. Consequently, establishing a photocatalytic process using clean and renewable solar energy would require the improvement of photocatalysts to enhance their visible light absorption. In this context, manganese oxides, whether supported or not on another material, could play a key role [41].

Manganese oxides have attracted attention in photocatalytic processes for the treatment of recalcitrant contaminants for their different morphologies and crystalline structures (α-, β-, γ- or δ-MnO_2_), in addition to their high efficiency, abundance, affordability (approximately 75% less expensive than TiO_2_) [29], high reproducibility during the manufacturing process, good adsorption properties, low toxicity, acid resistance, redox potential, and environmental friendliness [42,43,44]. The α-MnO_2_ phase has demonstrated a higher potential for removing organic compounds than the phases β-, γ-, and δ-MnO_2_, because α-MnO_2_ has the lowest oxidation state of all phases, according to Nawaz et al. [45]. Therefore, the α-MnO_2_ phase has the highest catalytic activity, owing to its high redox power. MnO_2_ has, as a function of the structure resulting from the synthesis process, a forbidden band energy of approximately 1–2 eV, which enables its activation in the visible region and makes it a highly competitive catalyst among other classical oxides [46,47]. Furthermore, manganese oxides can improve their efficiency by supporting them on carbonaceous materials [48,49,50]. As a result, modifying materials such as granular activated carbon (GAC) with manganese oxides (see Table S1) could lead to a better photocatalysis performance for ANI and BTH removal. For more details see Supplementary Materials.

In recent studies comparing different types of reactors, the authors have concluded that integrating an AOP with packed-, fixed-, and fluidised-bed (FBR) reactors facilitates large-scale operations and requires less oxidant and catalyst loading to degrade pollutants [51]. Nevertheless, Tian et al. [52] and Bello et al. [1] concluded that packed- and fixed-bed reactors were not suitable for photocatalytic processes, because they cannot provide a great surface area and mass transfer, and only a small fraction of the catalyst is exposed to light. However, fluidised beds, which are widely used in other petrochemical, combustion, or gasification applications would overcome these disadvantages by providing a good mass transfer rate and uniform mixing of the liquid-solid (LS) contact, a robust design for alterations in the starting effluent, a large-volume water-processing capacity, and a low operating cost. Moreover, fluidised beds are easy to operate, scale up, and build [5,53].

Most published studies have demonstrated improvements in the performance of processes which apply FBRs. Kanki et al. [54] studied the photocatalytic degradation of phenol and bisphenol A in an FBR using TiO_2_-coated ceramic particles, reporting that under the same oxidation conditions, the contaminant was removed four times faster in this FBR than in other reactor configurations. Huang and Huang [55] achieved 98% mineralisation of an aqueous stream containing phenol with an FBR-photocatalytic system using FeOOH on glass spheres. In both cases, they concluded that the FBR system adapted to a photocatalytic process improved light penetration and contaminant adsorption on the materials.

Therefore, using a α-MnO_2_ catalyst supported on granular activated carbon (GAC) in a photocatalytic FBR could achieve high removal and mineralisation rates, as well as facilitate scaling up the process to the industrial level for treating industrial effluents containing recalcitrant contaminants such as ANI and BTH, using an inexpensive, energy-efficient, environmentally friendly, regenerative, and robust process with visible light.

In this work, we propose the implementation on a pilot scale of an efficient photocatalytic system with a novel MnO_2_/GAC catalyst operating in an FBR in order to remove aniline and benzothiazole from an industrial effluent from the accelerator production for rubber manufacturing. Such a system requires the development of a new catalytic material supported on GAC, Hydrodarco^®^ 3000 (HD 3000, Boston, MA, USA), using a hydrothermal technique with low environmental impact and high reproducibility to prepare the alpha crystalline phase, and adequately distributing α-MnO_2_ on the GAC surface. The MnO_2_/GAC catalyst in the FBR system will avoid any subsequent separation operation. In addition, we studied operational parameters, such as pH and catalyst loading, to show the potential of this global strategy based on the FBR-photocatalytic system for subsequent scaling-up to an industrial level and increasing mineralisation yields and recalcitrant compound degradation with the lowest environmental impact and economic and energy costs.

## 2. Materials and Methods

### 2.1. Chemicals

Hydrochloric acid (HCl, Merck, 37%, Darmstadt, Germany), sodium hydroxide (NaOH, Panreac, 50%, Barcelona, Spain), potassium permanganate (KMnO_4_, Probus, >99%, Esparreguera, Spain), sulphuric acid (H_2_SO_4_, Sigma-Aldrich, 98%, St. Louis, MI, USA), hydrogen peroxide (H_2_O_2_, Labkem, 30%), sodium bicarbonate (NaHCO_3_, Merck, >99.7%, Darmstadt, Germany), sodium carbonate (Na_2_CO_3_, Merck, ≥99.5%, Darmstadt, Germany), sodium chloride (NaCl, VWR, 99.8%, Radnor, PA, USA), dichloromethane (CH_2_Cl_2_, Merck, >99.9%, Darmstadt, Germany), and diphenylamine (C_12_H_11_N, Merck, 99%, Darmstadt, Germany) were used. Deionised water was supplied by a Milli-Q^®^ water purification unit. Hydrodarco^®^ 3000 granular activated carbon was supplied by Cabot Corporation (Boston, MA, USA).

### 2.2. Catalyst Preparation

The MnO_2_/GAC material was synthesised using a hydrothermal preparation technique. This method was selected due to its simplicity, good reproducibility, and high reliability. In addition, the size and morphology of the nanostructure could be easily adapted to the granular activated carbon support.

The activated carbon was pre-treated with a piranha solution consisting of a (70:30 *v*/*v*) mixture of H_2_SO_4_ and H_2_O_2_ for 24 h to remove any impurity derived from the GAC manufacturing process and to prepare the GAC surface for active phase deposition [56]; subsequently, washing with deionised water and drying in an oven at 80 °C were carried out. Then, 9.97 g of KMnO_4_ was dissolved in 228.36 mL deionised water and shaken for 5 min. Then, 21.19 mL of concentrated HCl was poured on the permanganate solution dropwise for 15 min. The minimum amount of HCl required was used to prevent the potential loss of crystallinity in the final product and unwanted oxides such as hausmannite [57]. During the synthesis, at room temperature, the following reaction was performed:

(1) $${2\;\mathrm{KMnO}}_{4}\left(\mathrm{aq}\right)+8\;\mathrm{HCl}\;(\mathrm{aq})\to {2\;\mathrm{MnO}}_{2}\;(s)+2\;\mathrm{KCl}\;(\mathrm{aq})+{3\;\mathrm{Cl}}_{2}{\;(g)+4\;H}_{2}O\;(l)$$

where in the main reaction by-product is KCl. Then, 14.0 g of pre-treated GAC was added to this solution under slow stirring for 1.25 h. Then, the mixture was transferred to a Teflon^®^ autoclave reactor and remained at 180 °C for 12 h in an oven. The resulting MnO_2_/GAC-3 composite was washed several times with deionised water until the removal of the excess of MnO_2_ and unwanted by-products and dried in an oven at 80 °C for 12 h. The MnO_2_/GAC-1 and MnO_2_/GAC-2 catalytic materials were prepared with 1.94 g and 18.41 g of KMnO_4_, respectively, corresponding to the stoichiometric ratio between KMnO_4_ and HCl, according to Equation (1). The MnO_2_ nanoparticles (NPs) were synthesised following the procedures used to prepare the MnO_2_/GAC-3 composite, without incorporating the activated carbon support.

### 2.3. Analyses

#### 2.3.1. Chemical Analysis

ANI and BTH removal was quantified by gas chromatography (GC) using an Agilent 6890N gas chromatograph (Agilent, Santa Clara, CA, USA) coupled to an Agilent 5975 mass spectrometer (Agilent, Santa Clara, CA, USA). The chromatograph was equipped with a 30.0 cm non-polar phase column.

The aqueous samples were adjusted to pH = 11.0 for ANI and to pH = 3.0 for BTH. The samples were prepared by extraction with CH_2_Cl_2_ (using 0.1% C_12_H_11_N as an internal standard). A sample volume of 0.4 µL was injected using the method described by Ferreiro et al. [21]. The degree of mineralisation was quantified by total organic carbon (TOC) analysis on a Shimadzu TOC-VCSH analyser with ASI-V autosampler (Izasa Scientific, Alcobendas, Spain). The colour was analysed using a PerkinElmer Lambda 10 UV–Vis spectrophotometer (PerkinElmer, Madrid, Spain) by directly measuring the absorbance at 455 nm, and the aromatic ring rupture was determined from the sample’s absorbance at 254 nm [58]. The turbidity was analysed using the turbidimeter Eutech TN-100 (Thermo Scientific, Singapore), and the chemical oxygen demand (COD) concentration was measured using Merck Spectroquant^®^ kits (Merck KGaA, Darmstadt, Germany). Conductivity, temperature, dissolved oxygen, and pH were measured using a PCE-PHD1 multiparameter meter (PCE Ibérica, Tobarra, Spain). Total ammonia was determined by YSI TruLine ion-selective electrode (Yellow Springs, OH, USA). The analytical methods used to characterise the industrial influent are detailed in the Supplementary Materials.

#### 2.3.2. Catalytic Material Characterisation

The catalytic material composite was characterised using several techniques to analyse both its physical and chemical properties. The morphology and microstructure of the smallest particles in each sample were analysed by performing high-resolution transmission electron microscopy (HRTEM) on a Philips CM200 microscope (Philips, Eindhoven, The Netherlands), with a LaB_6_ filament as an electron source operating at an acceleration voltage of 200 kV. The microscope was coupled to an EDAX Genesis 4000 energy dispersive X-ray (EDX) spectroscopy platform (AMETEK GmbH, Weiterstadt, Germany) with a Si(Li)-type detector with a Super Ultra-Thin window to analyse the chemical composition of both pristine GAC Hydrodarco^®^ 3000 and MnO_2_/GAC-3 composite. Spectra and images were acquired using the software EDAM IV (AMETEK GmbH, Weiterstadt, Germany). The samples were dispersed in an equimolar ethanol–water mixture and ultrasonicated. Observations of the surface morphology of the smallest particles of the composite and MnO_2_ nanoparticles were carried out on a MULTIMODE 8 atomic-force microscope (AFM) Nanoscope V Bruker (Azbil Telstar Technologies, Terrassa, Spain). In addition, scanning electron microscopy (SEM) using a JEOL JSM-7000F electron microscope (JEOL B.V., Nieuw-Vennep, the Netherlands) was used.

MnO_2_/GAC-3 was characterised in a Malvern Panalytical Mastersizer 3000 particle-size analyser (Malvern Panalytical, Malvern, UK). Other physical properties, such as the pore size or specific surface area, were determined through adsorption–desorption analysis with high-purity nitrogen (N_2_) at boiling temperature (−196 °C), using the Micromeritics Surface Area and Porosimetry System (ASAP) 2010 high-performance gas adsorption analyser (Micromeritics France S.A., Verneuil Halatte, France). Prior to the adsorption experiments, the sample was degassed at 383 K for 3 h. The pore-size distribution of the new catalyst was obtained by the BJH method [6].

The crystalline phase and crystal size were determined by X-ray diffraction (XRD) on a Philips PW1710 diffractometer (Philips, Eindhoven, the Netherlands). The samples were finely ground and subjected to CuKα radiation in continuous scan from 5° to 70° and at a 2θ angle sweep speed of 0.026° s^−1^. The data were analysed using the software Winplotr^®^ (Institut des Sciences Chimiques de Rennes, Rennes, France). The phases of the catalyst were identified by matching each characteristic peak with the Joint Committee on Powder Diffraction Standards (JCPDS) files.

The MnO_2_ content was measured by X-ray fluorescence (XRF) spectroscopy. From each sample, a borated glass bead was prepared by melting in an induction micro-furnace, the Spectromelt A12 flux (Merck KGaA, Darmstadt, Germany), with the sample using a 20:1 ratio. An oxidising agent was added to favour the elimination of all organic carbon and the fixation of the inorganic oxides. The chemical analysis of the beads was performed under a vacuum atmosphere using a Panalytical AXIOS wavelength-dispersion XRF sequential spectrometer (WDXRF). The fluorometer is equipped with an Rh tube and three detectors (gas flow, scintillation, and Xe seal) (Malvern Panalytical, Malvern, UK).

The dispersion of the deposited metallic active phase MnO_2_ on GAC was determined by H_2_ chemisorption in a Micromeritics ASAP 2020 Plus analyser (Micromeritics France S.A., Verneuil Halatte, France). The reduction was performed by degassing 0.35 g of catalyst at 300 °C for 60 min, followed by reduction at a 50 mL min^−1^ flow with a 5% H_2_/Ar gas stream at 350 °C for 2 h. The adsorption isotherm was performed with H_2_ at 35 °C. To assess the amount of chemisorbed H_2_, the adsorption isotherm was repeated, gassing the sample again for 60 min, thereby determining the difference between the first and second isotherms.

Surface functional groups of pristine GAC and MnO_2_/GAC composite samples were identified using Fourier transform infrared spectroscopy (FTIR). Samples were ground in an agate mortar, and the resulting powders were mixed with anhydrous KBr. A pressed disc of the mixed sample was placed in a disc holder in a JASCO 4200 spectrometer (JASCO Corporation, Tokyo, Japan) equipped with a deuterated L-alanine doped triglycene sulphate detector (DLATGS). Using Spectra Manager software V 2.14.02 (JASCO Corporation, Tokyo, Japan), spectra were acquired in transmittance mode in the range 4000–400 cm^−1^ with an average of 64 scans and at a resolution of 4 cm^−1^. A pressed disc of pure KBr was used as a background for each measurement.

The chemical state of MnO_2_ deposited on the GAC support was determined by X-ray photoelectron spectroscopy (XPS) on a Leybold-Heraeus LHS-10 spectrometer (Leybold GmbH, Munich, Germany), with a twin anode Al/Mg X-ray source and a hemispherical electron energy analyser (HSA). XPS measurements were performed with a 150 W X-ray source, taking the C_1s_ peak (284.8 eV) as reference with an uncertainty of 0.2 eV. The samples were prepared on double-sided adhesive tape, compatible with high vacuum, and the XPS data were fitted using the XPSPEAK41 program (Leybold GmbH, Munich, Germany). Samples were measured in duplicate. Peaks’ deconvolution was performed using a nonlinear Shirley baseline to subtract the background, as well as a combination of Gaussian and Lorentzian type curves.

The point of zero charge (PZC) was determined using the method described by Ferreiro et al. [6] on a NaCl solution with 0.100 g of composite, measuring the pH on a CRISON GLP 22 pH meter (Hach Lange Spain, L’Hospitalet de Llobregat, Spain).

The optical properties to calculate the band gap of the new materials were characterised by UV–Vis absorption spectroscopy (200–2200 nm) at 25 °C on the Agilent Cary 5000 UV–Vis–NIR spectrophotometer (Agilent, Santa Clara, CA, USA).

### 2.4. UV-A Pilot Plant Description and Experimental Procedure

**Figure 1:**
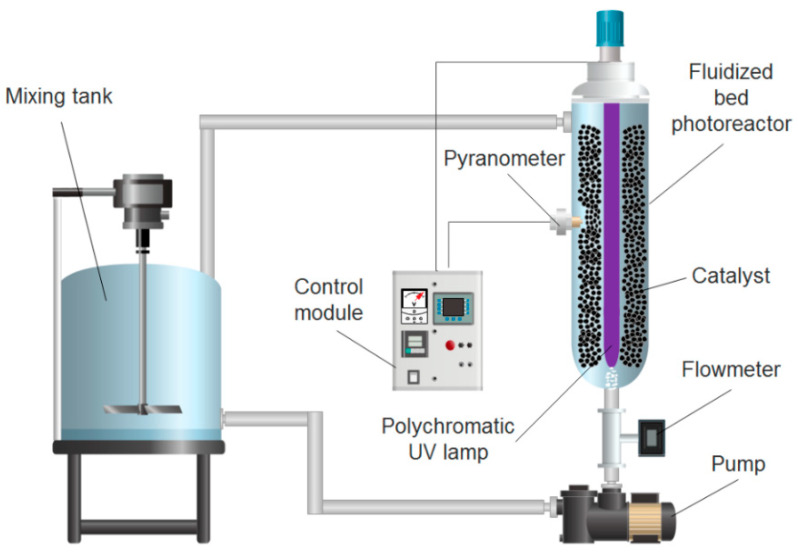
Schematic representation of the FBR-photoreactor at pilot scale used in the photocatalytic and adsorption experiments for aniline and benzothiazole removal from industrial effluents.

Aniline and benzothiazole contained in industrial effluents were removed on an FBR-photoreactor at pilot scale AOP 1 of h2o.TITANIUM^®^ (patent US20030059549A1) [59], which was modified for fluidised bed operation (see Figure 1).

The pilot plant consisted of a 47.3 cm long, 2.15 L stainless steel tubular reactor with a 25 W polychromatic UV-A lamp (BL368/4-Eubizz Water, Eubizz Water, Høyanger, Norway) (see Figure S1), protected by a quartz tube (Ø_int_ = 3.7 cm) and positioned axially in a cylindrical stainless steel photoreactor (Ø_int_ = 7.6 cm). The pilot plant was operated in batch mode with a recirculation flow rate of 0.75 m^3^ h^−1^ supplied by a 0.18 kW centrifugal pump (Pool Pump-72512, The Pool Shop, Tauranga, New Zealand) and a total volume of 10 L. The flow was controlled with a flow meter (GPI Electronic Digital Meter, Sparta, NJ, USA).

The irradiated surface of the FBR-photoreactor (*S*_irradiated_, m^2^) and the irradiated liquid volume (*V*_irradiated_, m^3^) were estimated using Equations (2) and (3):

(2) $$S_{\mathrm{irradiated}}=2\times \pi \times r_{\mathrm{int},\;\mathrm{FBR}}\times L_{\mathrm{lamp}}$$

(3) $$V_{\mathrm{irradiated}}=L_{\mathrm{lamp}}\times \pi \times \left(r_{{}_{\mathrm{int},\;\mathrm{FBR}}}^{2}-r_{\mathrm{int},\;\mathrm{lamp}}^{2}\right),$$

where *L*_lamp_ is the length of the UV-A lamp (m), and *r*_int, FBR_ and *r*_int, lamp_ are the inner radio of the FBR and inner UV-A lamp (m), respectively. The irradiated area was 0.05 m^2^, with an irradiated volume of 1.63 L. To compare the energy consumption with other reaction systems such as slurry reactors, the accumulated UV-A energy per unit of volume (*Q*_UVA_, kJ L^−1^) was calculated using Equation (4):

(4) $$Q_{\mathrm{UVA}}=Irradation\;dose\times \frac{S_{\mathrm{irradiated}}}{V},$$

where the *Irradiation dose* (kJ m^−2^) is the product of the irradiance emitted by the UV-A lamp in W m^−2^ multiplied by the exposure time in seconds. The irradiance emitted by the lamp was monitored throughout the experiment using a radiometer (PLS Systems AB, Sloga Ingenieros S.L., Puertollano, Spain). Under favourable operating conditions, a maximum irradiance of 155.8 W m^−2^ was reached, which corresponds to a *Q*_UVA_ value of 13.04 kJ L^−1^.

The photocatalytic experiments were performed at a constant temperature of 26 °C, a constant recirculation flow rate, and constant pH, with a variation of less than 3% in the averaged physicochemical characteristics of the industrial effluent outlined in Table 1.

The effluents were introduced into a mixing tank equipped with a mechanical stirrer until complete homogenisation (10 min), adjusting the initial pH. An initial sample was taken to verify the ANI and BTH concentrations. Subsequently, the dose of catalyst was added, and the recirculation system was connected until it reached the adsorption equilibrium (0.12 h). Then, the UV-A lamp was turned on, and the photocatalytic experiment started. The samples were collected at regular intervals to analyse aniline and benzothiazole degradation, as well as other physicochemical parameters. All experiments were performed in triplicate, with an error lower than 5.3%. Similarly, all collected samples were filtered with a 0.45-µm MF Millipore filter (Merck KGaA, Darmstadt, Germany) prior to their analysis.

## 3. Results and Discussion

### 3.1. Catalyst Characterisation

**Figure 2:**
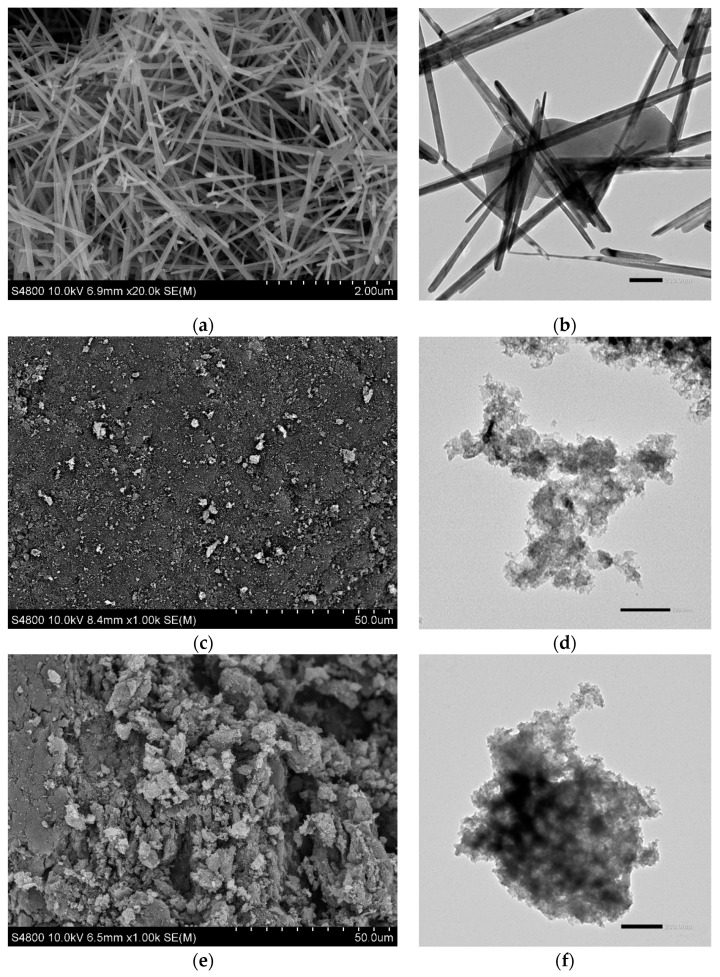
SEM (**left**) and HRTEM (**right**) micrographs of structures: (**a**,**b**) α-MnO_2_ nanoparticles; (**c**,**d**) commercial activated carbon Hydrodarco^®^ 3000; (**e**,**f**) synthesised MnO_2_/GAC-3 composite.

The type of composite structure used in the photocatalysis process may determine the results. Figure 2 shows SEM and HRTEM images of powder MnO_2_, GAC Hydrodarco^®^ 3000, and MnO_2_/GAC-3 structures.

Figure 2a,b show that the α-MnO_2_ phase has a morphology typical of a basic octahedral structure in the shape of needles, which are approximately 3 mm long and 50 to 250 nm wide, with a smooth texture, similar to that observed by Nawaz et al. [45] in a comparative study conducted with different manganese oxide phases. As shown in Figure 2b, defects in the amorphous structure could trap excited electrons, prolonging the separation of charge carriers, which some authors have related to an increase in the organic pollutant degradation efficiency [29]. However, the formation of particles agglomeration was observed, which could lead to a reduction in the number of catalyst active sites available for the photocatalytic reaction.

Figure 2c,d show the typical structure of microporous granular activated carbon with highly rough edges, irregular cavities, and fine open pores, similar to that observed by Alhamed et al. [60]. In contrast, the images in Figure 2e and f show spongy α-MnO_2_ nanoparticles that are approximately 5 µm in size and are responsible for increasing the external surface and the interior of the pores. In addition, the nanoparticles were well-dispersed, which increased the heterogeneity of the photocatalyst surface and helped to develop a porous structure without agglomerated particles, thus preventing the blockage of the pores and consequently reducing the catalytic and adsorptive properties of the new composite. Semi-quantitative elemental analysis of an EDX spectrum indicated the presence of Mn and C (see Figure S2).

Recently, Zhou et al. [50] doped ε-MnO_2_ samples with graphene, preparing a non-homogeneous material with a specific surface 24.3% higher than that of the initial ε-MnO_2_.

**Figure 3:**
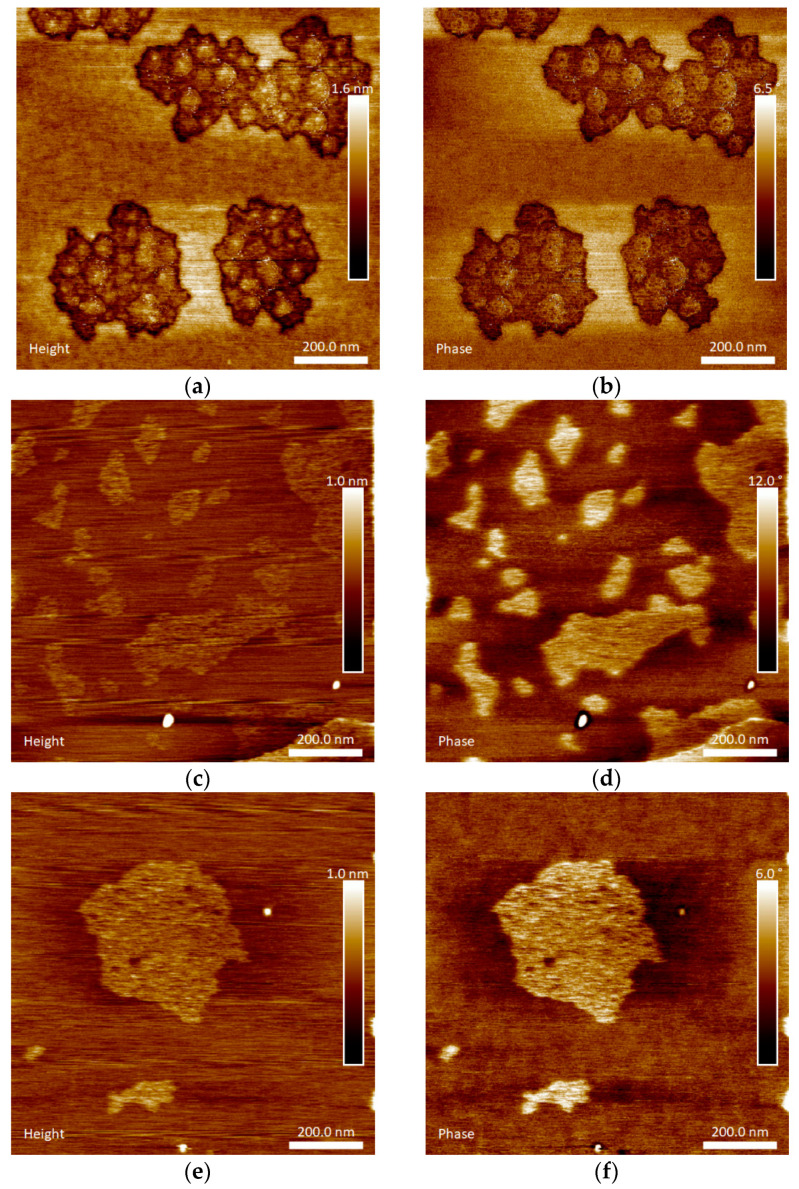
AFM topographic images of (**a**,**b**) α-MnO_2_ nanoparticles; (**c**,**d**) commercial activated carbon Hydrodarco^®^ 3000; (**e**,**f**) synthesised MnO_2_/GAC-3 composite.

Figure 3 shows AFM images of α-MnO_2_ nanoparticles, GAC Hydrodarco^®^ 3000, and the MnO_2_/GAC-3 composite. This technique revealed particles of 250 nm.

The morphological study of Figure 3a,b shows that α-MnO_2_ nanoparticles were spherical, with a diameter of 30–40 nm, according to topographic images, forming large structures consisting of 200 nm agglomerates. This behaviour was similar to that observed by Khan et al. [61] after synthesizing α-MnO_2_ nanoparticles using a precipitation method. In turn, Figure 3c,d show that commercial GAC had a rather uniform surface with small irregularities of approximately 0.5 nm, which were attributable to the porosity of the activated carbon. Finally, Figure 3e,f show that the particles were adequately dispersed on the surface of the GAC. Figure 3e shows changes in the 1 nm surface, thus confirming the MnO_2_ nanocrystals observed in HRTEM images. In addition, the comparison of the image of phase changes in Figure 3d–f indicates that the synthesised composite material MnO_2_/GAC-3 acquired significantly different properties. The MnO_2_/GAC composite synthesis method proposed in this study represents a major development compared with other procedures, such as that of Ma et al. [41], because the MnO_2_/GAC composites were prepared by adsorption from solution. As a result, MnO_2_ homogeneously covered the surface of the activated carbon. According to the SEM images, the agglomerates practically blocked and rendered useless the internal porosity of activated carbon, thus decreasing the ability of the composite to adsorb organic compounds [4,20].

**Figure 4:**
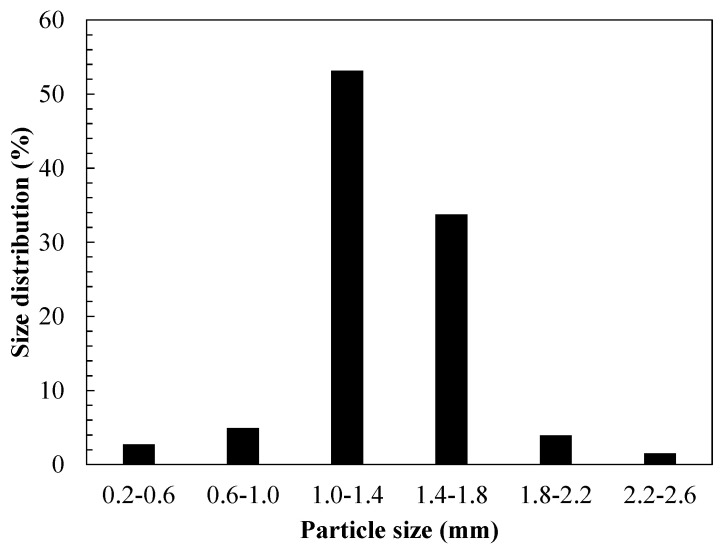
Particle-size distribution of the synthesised MnO_2_/GAC-3 composite.

The size of the activated carbon particles modified with MnO_2_ is a relevant property required for the operation of an FBR [54]. The Geldart classification is one of the most useful ways of classifying solids [1], and it is usually applied to an FBR in group B (with a size between 100 and 800 µm) and group D (larger than 1 mm) particles [62]. Figure 4 shows the particle-size distribution assessed by laser diffraction spectrometry.

As shown in Figure 4, the average particle diameter was 1.3 mm, Geldart group D, with a mean standard deviation of 0.15. Fernández et al. [63] assessed particle-size effects on dissolved organic carbon (DOC) by comparing two size ranges of zeolitic supports (0.2–0.5 mm and 0.5–0.8 mm, respectively).

**Figure 5:**
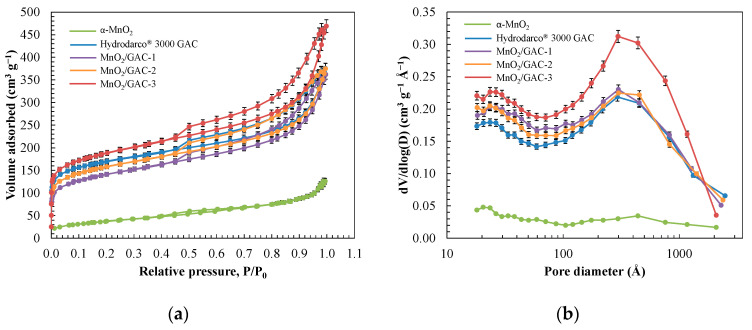
N_2_ adsorption isotherm (**a**) and pore-size distribution (**b**) of synthesised α-MnO_2_ nanoparticles, commercial activated carbon Hydrodarco^®^ 3000, and three MnO_2_/GAC composites synthesised in this study.

Figure 5 shows the adsorption–desorption isotherms as well as the hysteresis curve and pore-size distribution, which were calculated from the desorption data of the studied materials.

Figure 5a shows that the initial amount of adsorbed N_2_ rapidly increased until it attained a relative pressure of P/P_0_ = 0.2 before reaching GAC and MnO_2_/GAC composite saturation. The isotherm of the composites, which was typical of microporous materials, was associated with a type IV isotherm in which adsorbed N_2_ formed a monolayer, while the second branch formed multiple layers. All four cases showed an H3 hysteresis loop, which was associated with capillary condensation that occurs in the mesopores [64]. Conversely, α-MnO_2_ nanoparticles showed a type II isotherm, which is commonly found in low-porosity or macroporous materials. The pore-size distribution of the composites and GAC ranged from 200 to 500 Å, while the α-MnO_2_ nanoparticles ranged from 20 to 1000 Å. This wider range may have resulted from the temperature applied during the hydrothermal synthesis process, which was high enough to enable some degree of sintering and thus obtain a practically homogeneous distribution for a wide range of sizes [65].

Table 2 outlines the physical properties of the new composites, α-MnO_2_ nanoparticles, and GAC Hydrodarco^®^ 3000. The results of Table 2 showed an increase (9.47%) in the Brunauer, Emmett, and Teller (BET) specific surface area of the MnO_2_/GAC-3 composite, which was 664.1 m^2^ g^−1^, in comparison to the value of 601.2 m^2^ g^−1^ for GAC Hydrodarco^®^ 3000. The external surface area increased from the initial 276.4 m^2^ g^−1^ for GAC Hydrodarco^®^ 3000 to 345.49 m^2^ g^−1^ for the MnO_2_/GAC-3 composite. This increase was in agreement with topographic AFM images showing that the roughness of the MnO_2_/GAC-3 composite significantly increased, and thus matching the HRTEM and SEM images in which the MnO_2_/GAC-3 composite displayed a good distribution of MnO_2_ nanoparticles. In addition, the activation of the MnO_2_/GAC-3 composite by mild calcination opened the pores, increasing the mean pore diameter (88.03 Å), as described by Ghasemi et al. [66] and Ferreiro et al. [6]. In turn, a major drawback was observed in the composites MnO_2_/GAC-1 and MnO_2_/GAC-2, because MnO_2_ nanoparticles formed agglomerates and blocked the internal porosity of GAC.

The results of the MnO_2_/GAC-3 composite represent an improvement over previously published preparation methods. For example, Liu et al. [67] synthesised MnO_2_/GAC composites by electrodeposition to be used as an electrode with a smaller surface (625 m^2^ g^−1^) compared with the original activated carbon (724 m^2^ g^−1^). Choi et al. [68] synthesised composites with various MnO_2_ proportions using a simple hydrothermal preparation technique, which led to a drastic decrease in specific surface area from the 1844 m^2^ g^−1^ of the original activated carbon to 1017 m^2^ g^−1^ of the MnO_2_/GAC composite, with a 12.37% MnO_2_ content. Yang et al. [69] managed to keep the physical properties of activated carbon unchanged. In both cases, the authors highlighted that MnO_2_ deposition on GAC partly blocked the carbon pores, thereby decreasing the specific surface area, and consequently causing the loss of adsorptive properties of the activated carbon.

The crystallography of photocatalysts plays a key role in the optical, physical, and chemical properties of these materials. Reddy et al. [23] made considerable efforts to control the size of the synthesized nanoparticles, as well as the size of the crystalline phase, both related to minimising the recombination of electron-hole pairs, the energy band gap, and the surface area. Preparing a phase without impurities, such as α-MnO_2_, may help to improve the interaction between the photocatalyst and the light source.

**Figure 6:**
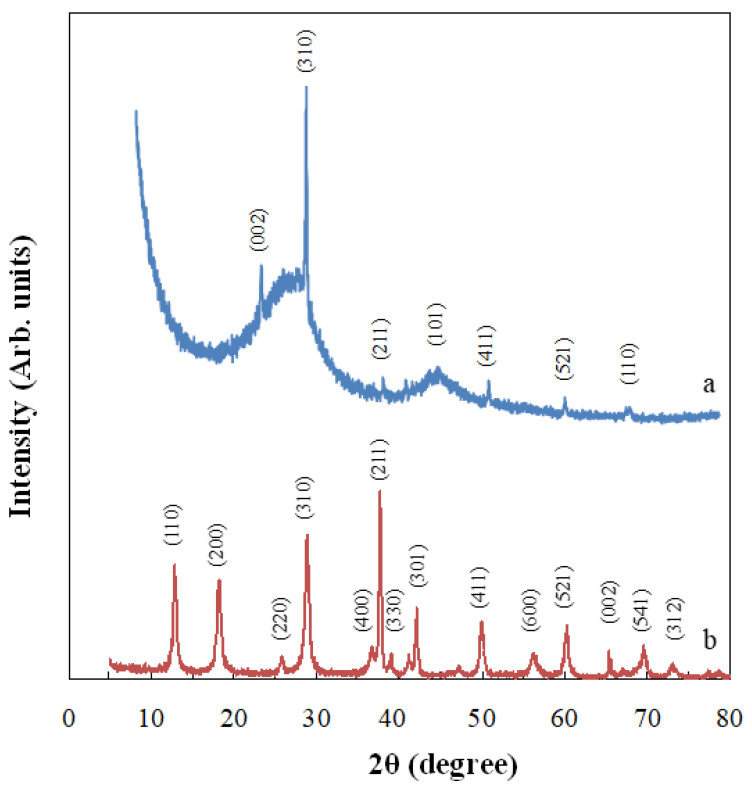
XRD spectra of the MnO_2_/GAC-3 composite (**a**) and synthesised MnO_2_ powder (**b**).

Figure 6 shows the XDR spectra of the MnO_2_/GAC-3 composite and MnO_2_ nanoparticles prepared by hydrothermal synthesis. Figure 6a shows the typical diffraction peaks at the positions 2θ = 21.2, 25.5, 36.6, 43.7, 50.3, 60.2, and 68.4°, corresponding to an amorphous carbon structure (JCPDS file No. 75-1621) and a tetragonal structure of α-MnO_2_ (JCPDS file No. 44-0141). Figure 6b shows diffraction peaks at the positions 2θ =12.8, 18.1, 25.5, 28.6, 36.6, 37.6, 38.9, 42.1, 50.3, 56.2, 60.2, 65.4, 69.5, and 73° corresponding to a tetragonal structure of α-MnO_2_ (hollandite). According to Thackeray [70], this crystalline structure was made of double MnO_6_ octahedra in which the α-MnO_2_ structure tied at the edges to form 1 × 1 and 2 × 2 tunnels of 1.89 and 4.6 Å, respectively.

The spectra in Figure 6 show that introducing MnO_2_ in the activated carbon structure had no effect on the catalyst structure with respect to the formation of α-MnO_2_. However, the intensity of the characteristic peaks of α-MnO_2_ was attenuated, which may be due to changes in crystallinity resulting from GAC incorporation.

The average size of the crystals, *D* (nm), was determined from crystallographic data using the Debye–Scherer equation [71]:

(5) $$D=\frac{k\times \lambda }{\beta \times \cos\theta },$$

where in *k* is the Scherer constant (0.94), λ is the length of the X-ray source (for Cu Kα, λ = 0.15418 nm), *β* is the angular width based on the full width at half maximum (FWHM) of the peak, and *θ* is the diffraction angle. According to Shen et al. [72], the crystal size may introduce some error in the determination of *β* owing to stress effects of the crystal and the instrument used to record the spectra. For this reason, the FWHM was corrected by widening the instrumental line, *b*, thus recalculating the *β* value using Equation (6):

(6) $$\beta =\sqrt{FWHM^{2}-b^{2}}$$

Equations (5) and (6) were used to estimate the theoretical average crystal size of α-MnO_2_ nanoparticles (2.68 nm) and the MnO_2_/GAC-3 composite (12.79 nm). The average sizes of the MnO_2_/GAC-1 and MnO_2_/GAC-2 composites were 18.06 and 14.30 nm, respectively, owing to the formation of agglomerates, as previously mentioned [71].

**Figure 7:**
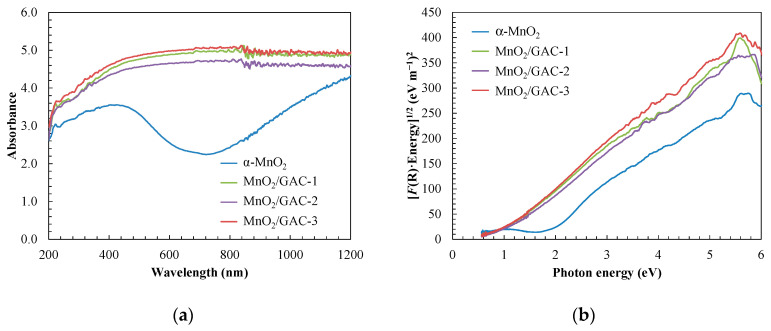
UV–Vis absorption spectra (**a**) and determination of the band gap values of the new catalysts (**b**).

Another relevant aspect of the new composite was the range of light necessary for its photoactivation. Figure 7a shows the absorption spectra of α-MnO_2_ nanoparticles and the three MnO_2_/GAC composites prepared in this study. α-MnO_2_ showed an intense absorption band in the UV region below 500 nm. This band may be due to charge transfer between the 2p orbital of oxygen and the 4d orbital of manganese [50].

The band gap was determined using the Tauc plot (Figure 7b) and the transformed Kubelka–Munk function [71,73]:

(7) $$\alpha \times h\times \nu =A\times {\left(h\times \nu -Bandgap\right)}^{n}$$

(8) $${\left[F\left(R\right)\times h\times \nu \right]}^{n}=A\times \left(h\times \nu -Bandgap\right)$$

(9) $$F\left(R\right)=\frac{{\left(1-R\right)}^{2}}{2\times R},$$

where *ν* is the vibration frequency (Hz), *A* is the absorbance (AU), *h* is the Planck constant (4.135 × 10^−15^ eV s), R is the diffuse reflectance (%), and *n* has a constant value of 0.5 for direct transitions. According to Figure 7b, the band gap of each photocatalyst was determined by representing Equation (8) as a function of the band gap and extrapolating to the abscissa axis in its linear region.

As shown in Figure 7b, the band gap decreased from 1.81󠆸 ± 0.03 eV in α-MnO_2_ nanoparticles to 0.90 ± 0.03 eV in the MnO_2_/GAC-1 composite, while the band gaps of MnO_2_/GAC-2 and MnO_2_/GAC-3 composites were 0.92 ± 0.03 and 0.95 ± 0.03 eV, respectively. Therefore, the composite material resulted in the nanoparticle absorption changing to longer wavelengths. Reducing the band gap made it possible to activate the new composites under visible light. Improving the absorbance of visible light of the new composites could improve photocatalytic activity, as more electron-hole pairs can be generated. Moreover, the addition of a carbonaceous material could inhibit the recombination of the photogenerated electron-hole pairs, as was previously reported in other oxides such as TiO_2_ and Zn or Al oxides with carbonaceous structures [50,71,73].

**Figure 8:**
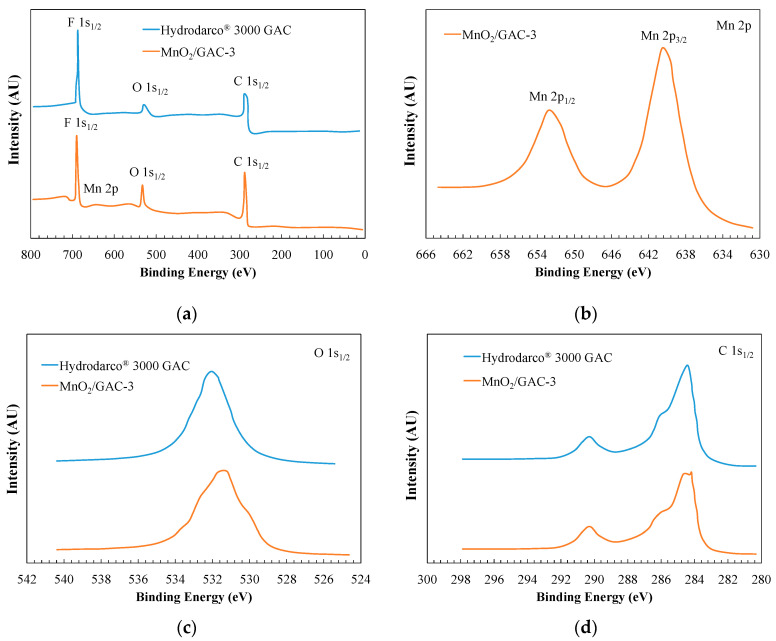
XPS spectra of the GAC Hydrodarco^®^ 3000 and the MnO_2_/GAC-3 composite (**a**) XPS; and enlarged spectra of the peaks: (**b**) Mn 2p; (**c**) O 1s_1/2_; (**d**) C 1s_1/2_.

The chemical elements and the oxidation state of the GAC Hydrodarco^®^ 3000 and the MnO_2_/GAC-3 composite were determined by X-Ray photoelectron spectroscopy (XPS). Figure 8 shows the resulting spectrum, confirming the presence of Mn, O, and C.

Figure 8c shows that the intensity of the peak corresponding to O 1s_1/2_ (530.3 eV) increased in the composite of GAC Hydrodarco^®^ 3000 with MnO_2_. This increase may be due to a possible change in the surface of the MnO_2_/GAC-3 composite in relation to the original GAC, resulting from the formation of a new functional group associated with some type of Mn–O–Mn bond [74]. Another relevant result in Figure 8b shows Mn 2p_1/2_ and Mn 2p_3/2_ peaks with 654.0 eV and 642.2 eV bond energies, respectively. Choi et al. [68] associated the Mn 2p_3/2_ peak with the oxidation state of Mn (IV) through the binding energy at which the peak was detected. According to Xiong et al. [75], preparing a strongly oxidising active phase of manganese in its oxidation state (IV) was crucial, because under this oxidation state, manganese deposited on GAC plays a key role in promoting the generation of hydroxyl radicals and therefore providing photocatalytic processes with a high performance of organic pollutant removal.

Regarding the detection of C 1s_1/2_, Figure 8d shows three types of bonds. At a 284.5 eV binding energy, the peak is associated with a C–C bond. The peak at 286.1 eV likely corresponds to a C–O bond, while the peak at 290.4 eV indicates a C=O bond. Therefore, no notable changes were observed between the original GAC and the MnO_2_/GAC-3 composite.

**Figure 9:**
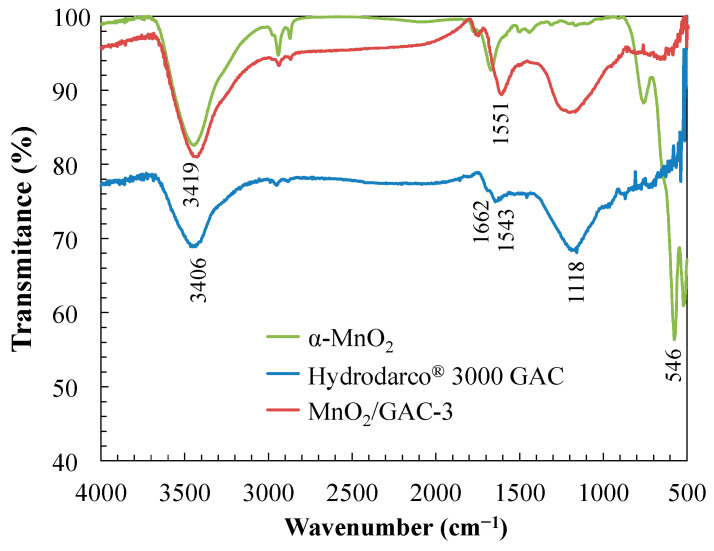
FTIR spectrum of the region between 4000 and 500 cm^−1^ of α-MnO_2_ nanoparticles, GAC Hydrodarco^®^ 3000, and MnO_2_/GAC-3 composite.

Figure 9 shows the FTIR spectrum of the synthesised α-MnO_2_ nanoparticles, the GAC Hydrodarco^®^ 3000, and the MnO_2_/GAC-3 composite.

The absorption band that was highlighted in Figure 9 in the 3100–3700 cm^−1^ range was associated with a bending vibration of the O–H bond, which would correspond to the water used to prepare the potassium bromide pellet [6]. The 1500–1700 cm^−1^ bands would be associated with stress vibrations of groups with double bonds such as C=O or their conjugated C–O bonds, as observed in the XPS spectra [76,77]. The band observed at 1118 cm^−1^ could correspond to a stretching vibration of the C–O bond. Finally, MnO_2_ was identified from the absorption band at 546 cm^−1^, which would correspond to a stretching vibration of the Mn–O bond. The comparison of the spectra of the GAC Hydrodarco^®^ 3000 and the MnO_2_/GAC-3 composite showed that during the process of MnO_2_ deposition, the GAC surface changed significantly, as was found by other researchers such as Ma et al. [74]. These changes were shown by the disappearance of the bands at 1543 and 1662 cm^−1^, which were replaced by a single peak red-shifted to 1551 cm^−1^.

To better understand the chemical properties of the new MnO_2_/GAC composite and their implications for the photocatalytic process, the point of zero charge (PZC), the degree of dispersion of the active phase MnO_2_ on GAC, and the MnO_2_ content were measured in the three MnO_2_/GAC composites synthesised in this study. Metal dispersion (*Disp*) was calculated based on the coating of the H_2_ chemisorption monolayer in MnO_2_/GAC according to Equation (10):

(10) $$Disp=\left(\frac{V_{\mathrm{mon}}}{22414}\right)\times S\times M\times \frac{100}{Metal\;content},$$

where *V*_mon_ is the volume of the H_2_ monolayer in MnO_2_/GAC in cm^3^ g^−1^, *S* is the stoichiometric factor of H_2_ to the Mn atom (*S* = 2) [78], *M* is the atomic weight of Mn in g mol^−1^ (*M* = 54.938 g mol^−1^), and *Metal content* (%) is the weight percentage of the deposited metal determined by XRF analysis. As shown in Figure S3, the amount of H_2_ chemisorbed on the catalyst surface was calculated as the difference between the first and the second isotherms.

Table 3 outlines the chemical properties of each sample. The results indicate that the MnO_2_/GAC-3 composite had the highest MnO_2_ content (3.78%). Although this active phase content was modest when compared to the amount of MnO_2_ deposited by Choi et al. [68] (MnO_2_ content of 12.37%) or by Liu et al. [67] (MnO_2_ content of 6.7%), the hydrothermal preparation method presented in this study was different, because the external surface of GAC was kept without obstructing its pores and channels and without compromising the amount of MnO_2_ deposited on GAC. These findings were confirmed by metal dispersion, because dispersion increased with the MnO_2_ content, with a 21.0% Mn dispersion in the MnO_2_/GAC-3 composite. In turn, in the composites with low Mn content, dispersion decreased as the surface area decreased. In the MnO_2_/GAC-1 and MnO_2_/GAC-2 composites, the decrease in Mn dispersion may have resulted from the formation of particle aggregates, which blocked carbon porosity [72].

The PZC of the new composites decreased slightly as the amount of deposited MnO_2_ increased, with a pH_PZC_ of 6.61 for MnO_2_/GAC-3. The surface change could influence the adsorption step in the photocatalytic removal of aniline and benzothiazole, because they have dissociation constant (pKa) values of 4.61 and 0.85, respectively [21].

Consequently, the MnO_2_/GAC-3 composite would have the best properties for an efficient use in an FBR-photoreactor.

### 3.2. Reaction in the FBR-Photoreactor

The application of an FBR in a photocatalysis process requires the careful determination of building and operating parameters. The lack of a clear and robust design method could result in an ineffective FBR implementation in water treatment processes on an industrial scale [63]. During its implementation, researchers often face common problems, such as insufficient fluidisation or pneumatic entrainment of the catalyst, reactor failures, and poor treatment due to a high dose of catalyst [53]. Consequently, not only the appropriate photocatalyst but also the correct design parameters are critical steps in correctly configuring the photoreactor. Therefore, the reactor geometry and surface velocity of the fluid are discussed below.

#### 3.2.1. FBR Sizing

Reactor size affects photocatalyst mixing and mass transfer. With regard to geometry, cylindrical reactors provide better mixing than those with rectangular geometry, because dead zones prevent reaction bulk homogenisation. To stabilise feed flow and to minimise eddies, backmixed zones, or sudden bed expansion due to highly turbulent flow, the fluid should enter through a uniform cross-sectional area in flat-bottom reactors [79]. Furthermore, a 5° angle in the fluid inlet mouth would ensure the minimisation of the problem of sudden bed expansion [54].

The aspect ratio between the height and the diameter of the FBR directly affects the flow velocity of the fluid and therefore the mixture between the liquid phase and the solid [5]. According to Bello et al. [1], a suitable aspect ratio for an FBR should be between 5 and 25 for a laboratory-scale installation and between 2 and 5 for a water treatment plant. Consequently, the FBR was built with an aspect ratio of 6.22, according to the optimisation by Ochieng et al. [80].

Furthermore, other elements, such as the UV-A lamp, acted as baffle elements to modify the flow and to improve the fluidisation of the MnO_2_/GAC-3 catalyst particles, which promoted a more uniform mixture throughout the reactor. In this regard, authors such as Nam et al. [81] used a drag tube and other elements within the FBR to improve phenolic removal efficiency.

#### 3.2.2. Catalytic Material and Surface Velocity of Fluid in the FBR

When assessing the catalyst particle-size effect on mass transfer and on fluid dynamic properties, the specific area of the catalyst bed after fluidisation (*a*_S_) should be considered, because a high *a*_S_ leads to more satisfactory results [82]. The term *a*_S_ of the FBR system, which was 2.88 m^−1^, was determined from Equation (11):

(11) $$a_{S}=\frac{6\times \left(1-\varepsilon \right)}{d\times F_{S}}$$

where *ε* is the bed porosity, *d* is the mean particle diameter (mm), and *F*_S_ is a shape factor (*F*_S_ = 1, for spherical particles). Together with the surface area, the density of catalyst particles strongly influences the surface velocity of the fluid. In this regard, dense particles, which consist of non-porous materials, will require a higher velocity than porous materials such as activated carbon [79,83]. Hence, there are advantages of using activated carbon as MnO_2_ catalytic support over denser α-MnO_2_ nanoparticles. Moreover, the surface velocity is proportional to the energy needs of the process. Consequently, that velocity should be slightly higher than the minimum fluidisation to minimise operational costs as much as possible [1]. The minimum fluidisation velocity (*U*_mf_) was determined according to Equation (12):

(12) $$U_{\mathrm{mf}}=16.5\times \frac{d^{2}\times \left(\rho_{S}-\rho \right)\times g}{\mu }$$

where *ρ*_S_ and *ρ* are the specific gravity of the MnO_2_/GAC-3 catalyst and water (g m^−3^), respectively, *µ* is the viscosity of water (g m^−1^ h), and *g* is the gravitational constant (m h^−2^). In this study, a velocity of 165.3 m h^−1^, which is similar to the minimum fluidisation velocity, was used in the FBR system. Delebarre et al. [84] also worked with a fluid velocity near the minimum fluidisation velocity. Working at a *U*_f_ much higher than the *U*_mf_ would lead to a shorter reaction time, albeit not offset by the higher operational cost.

### 3.3. MnO_2_/GAC Composite Testing in Photocatalysis for Aniline and Benzothiazole Removal from Wastewater

**Figure 10:**
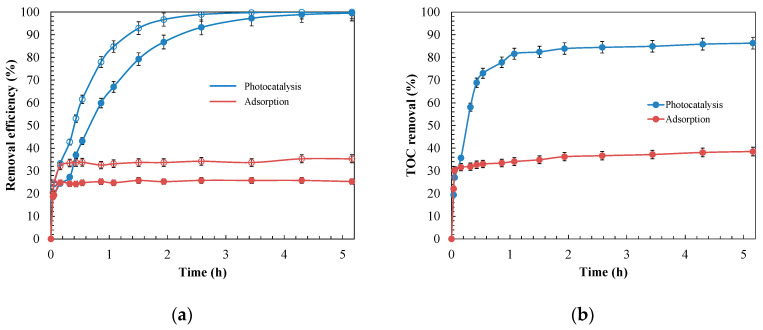
Removal efficiency of adsorption and photocatalysis processes for 12.0 mg L^−1^ of ANI (○) and BTH (●) at pH = 9.0 with 0.9 g L^−1^ composite MnO_2_/GAC-3. Evolution: (**a**) primary degradation; (**b**) mineralisation.

Activated carbon is the adsorbent most commonly used in the wastewater treatment industry for the effective retention of a broad spectrum of pollutants [85]. The removal performances of adsorption and photocatalysis processes with MnO_2_/GAC-3 composites were compared using an industrial effluent containing ANI and BTH. Figure 10 shows the evolution of the primary degradation of both contaminants and the total organic carbon (TOC) mineralisation.

Figure 10 shows that photocatalyst irradiation significantly improved ANI and BTH removal compared with the adsorption process, both in terms of primary degradation, completed after 5 h of irradiation, and in terms of mineralisation, which reached 86%, showing that MnO_2_/GAC-3 had photocatalytic activity. To determine the contribution of α-MnO_2_ deposited on GAC, the photocatalytic process and the adsorption capacity of the GAC Hydrodarco^®^ 3000, which was used as support to prepare the MnO_2_/GAC-3 composite, were compared. The results presented in Figure S4 indicate that UV-A irradiation of the GAC had no effect on aniline and benzothiazole removal or on the mineralisation, regardless of the pH of the solution. These findings confirm that GAC without α-MnO_2_ did not exhibit photocatalytic activity when using the light source.

**Figure 11:**
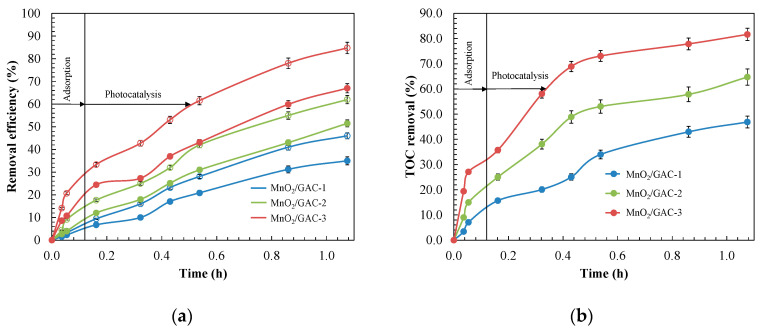
Comparison of the catalytic activity of MnO_2_/GAC composites. Evolution of the: (**a**) primary degradation of ANI (○) and BTH (●); (**b**) mineralisation. Experimental conditions: *C*_0_ = 12.0 mg L^−1^; pH = 9.0; *m*_CAT_ = 0.9 g L^−1^; *Irradiation dose* = 155.8 W m^−2^.

The catalytic activity of the composites prepared in this study is compared in terms of primary degradation and mineralisation in Figure 11.

As shown in Figure 11, the MnO_2_/GAC-3 composite had the highest degree of mineralisation (81.6%) and degradation of both ANI (84.7%) and BTH (67.0%) after 1.08 h of reaction. The presence of a higher α-MnO_2_ content (3.78%) in the MnO_2_/GAC-3 composite and the increase in BET surface area led to better removal results [86]. Crystal size could also influence the electron-hole recombination [87], thus generally decreasing the photocatalytic activity as the crystal sizes increased. Therefore, the MnO_2_/GAC-3 size of 12.79 nm was better than MnO_2_/GAC-1 (18.06 nm) and MnO_2_/GAC-2 (14.30 nm). The same trend was observed by Khlyustova et al. [86].

#### 3.3.1. Kinetics of ANI and BTH Adsorption and Photodegradation with the MnO_2_/GAC-3 Composite

To identify the controlling stage of the photocatalytic process, the degradation kinetics of ANI and BTH were modelled under both darkness (adsorption) and UV-A light (photocatalysis), fitting them to pseudo-first-order (Langmuir–Hinshelwood) [88] and pseudo-second-order (Lagergren) [89] kinetic models, using the following equations:

(13) $$\frac{dq_{t}}{dt}=k_{1}\times \left(q_{e}-q_{t}\right),$$

where *q*_e_ (mg g^−1^) is the adsorption capacity at equilibrium, *q*_t_ (mg g^−1^) is the amount of ANI or BTH adsorbed at a given time *t*, and *k*_1_ (h^−1^) is the pseudo-first-order kinetic constant; and

(14) $$\frac{dq_{t}}{dt}=k_{2}\times {\left(q_{e}-q_{t}\right)}^{2}$$

where *k*_2_ (g mg^−1^ h^−1^) is the pseudo-second-order constant. The kinetic parameters that fitted the data shown in Figure 10 are presented in Table 4. The correlation coefficients (*R*^2^) of the pseudo-second-order model were higher than 0.90, but they were low for the pseudo-first-order model (*R*^2^ ≈ 0.67). Consequently, the pseudo-second-order model was the most appropriate to describe the evolution of both adsorption and photocatalysis. Assuming that the global photocatalysis process consists of a series combination of adsorption steps followed by the reaction, the kinetics of the slowest step will control the global process [21,30]. Therefore, the adsorption step controlled the global process, because its kinetic constant was lower than that of the photocatalytic reaction.

Nevertheless, the process can be affected by other experimental variables, such as pH or catalyst dose [4], that will be studied in detail in the following sections.

#### 3.3.2. Effects of pH and Catalyst Dose during the Photocatalysis Process for ANI and BTH Removal with the Composite MnO_2_/GAC-3

The effects of the pH and catalyst dose on the photocatalytic activity of the process were compared by simplifying a Langmuir–Hinshelwood kinetic model to a pseudo-first-order kinetic model. This approach is widely used to describe photocatalytic processes with various organic pollutants [73,90,91]. The rate equation was:

(15) $$r=\frac{dC}{dt}=k\times \frac{K\times C}{1+K\times C+\sum_{i}K_{i}\times C_{\mathrm{int}}}\approx k_{\mathrm{app}}\times C,$$

where *r* is the oxidation rate of the pollutants to CO_2_ (mg L^−1^ h), *C* is the concentration of the reactants (mg L^−1^), *k*_app_ is an apparent first-order kinetic constant (h^−1^), *t* is the irradiation time (h), *k* is the kinetic constant of the reaction (mg L^−1^ h), *K* and *K*_i_ are the Langmuir adsorption constants for *C* and reaction intermediates (L mg^−1^), and *C*_int_ is the concentration of the various intermediate products of ANI or BTH degradation (mg L^−1^).

Another parameter which is frequently used to compare apparent first-order kinetics is the half-life time *t*_1/2_ (h) (Equation (16)) [91]:

(16) t12=ln2kapp

The pH can affect the overall photocatalytic process with the MnO_2_/GAC-3 composite. For the highest photocatalytic activity, the most favourable pH will depend on the surface charge of the catalyst and on the oxidation potential of the reaction. Consequently, throughout the experiments, the pH was controlled to be kept constant. As a general rule, according to Equations (17)–(19), the surface of the MnO_2_/GAC-3 catalyst would be positively charged when the pH of the dissolution was lower than the pH_PZC_. Conversely, the surface would be negatively charged when the pH values were higher than the pH_PZC_ [92].

Under acid pH conditions (pH < pH_PZC_):

(17) $${\mathrm{MnO}}_{2}/\mathrm{GAC}+H_{2}O\to \mathrm{Mn}{\left(\mathrm{OH}\right)}_{2}/\mathrm{GAC}+2{\mathrm{OH}}^{-}$$

Under neutral pH conditions (pH ≈ pH_PZC_):

(18) $${\mathrm{MnO}}_{2}/\mathrm{GAC}+H^{+}\to \mathrm{Mn}{\left(\mathrm{OH}\right)}_{{}_{2}}^{+}/\mathrm{GAC}$$

Under alkaline pH conditions (pH > pH_PZC_):

(19) $${\mathrm{MnO}}_{2}/\mathrm{GAC}+{\mathrm{OH}}^{-}\to {\mathrm{MnO}}^{-}/\mathrm{GAC}+H_{2}O$$

**Figure 12:**
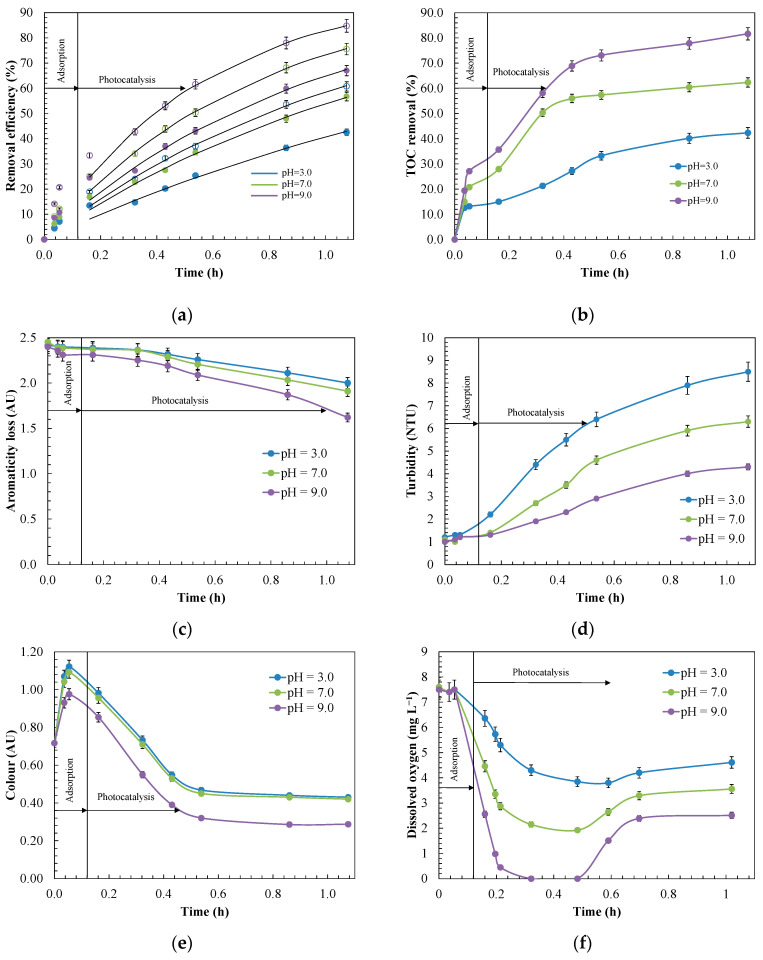
Study of the pH effect on the photocatalytic removal of ANI and BTH from industrial effluents with the MnO_2_/GAC-3 photocatalyst. Evolution of the: (**a**) primary degradation of ANI (○) and BTH (●) fitted to the apparent first-order kinetic model; (**b**) total organic carbon (TOC) removal; (**c**) loss of aromaticity; (**d**) turbidity; (**e**) colour induction during aniline and benzothiazole oxidation; (**f**) dissolved oxygen. Experimental conditions: *C*_0_ = 12.0 mg L^−1^; *m*_CAT_ = 0.9 g L^−1^; *Irradiation dose* = 155.8 W m^−2^; T = 26 °C.

The experiments were performed at pH 3.0, 7.0 and 9.0 to assess the effect on ANI and BTH degradation (Figure 12). The values of the kinetic constants (*k*_app_) and half-life time (*t*_1/2_) are outlined in Table 5. The experiments were properly fitted to the apparent first-order kinetic model with a correlation coefficient *R*^2^ higher than 0.98. For both mineralisation (81.6%) and degradation under UV-A light after 1 h of reaction, the most favourable conditions were found at a slightly basic pH (pH = 9.0) with half-life times of 0.39 h and 0.66 h for ANI and BTH, respectively. These conditions were favourable because the negatively charged MnO_2_/GAC-3 catalyst in alkaline medium contributed to electrostatic interactions with ANI and BTH, which were positively charged. David and Vedhi [93] reported a similar response to an alkaline pH in the degradation of cationic dyes. In addition, they observed that the generation of radical species increased as a consequence of the reaction between the hydroxide ions and the photogenerated holes.

In turn, at acid pH values (pH = 3.0), both the catalyst and ANI and BTH, which were positively charged, caused repulsion phenomena. Under these conditions, a more modest yield was assessed for both mineralisation (42.3%) and ANI (60.8%), as well as BTH (42.5%) removal. In addition, the half-life time of both ANI (0.79 h) and BTH (1.33 h) increased considerably, corresponding to *k*_app_ = 0.87 and 0.52 h^−1^, respectively [50].

Other variables, such as the formation of condensation compounds, including polyaniline, compared to other more easily oxidisable degradation intermediates, could explain the worst results at alkaline pH conditions [20,21]. In addition, Zhou et al. [25] reported that during BTH oxidation, organic radicals could react with each other by C–C coupling to generate dimers. These dimers in turn would react with BTH radicals to form macromolecules, which were difficult to oxidise.

Parameters such as the loss of aromaticity (Figure 12c) or turbidity (Figure 12d) could explain the differences in behaviour. Thus, the loss of aromaticity, which was expressed as the decrease in absorbance at 254 nm, corresponded to a greater degradation, owing to the breakdown of the aromatic structure. According to Sanchez et al. [94] and De Wever and Verachtert [13], at pH = 9.0, absorbance decreased further, owing to the possible formation of hydroxylated ANI intermediates such as resorcinol, catechol, *p*-benzoquinone, and carboxylic acids, or 2-mercaptobenzothiazole and 2-hidroxibenzothiazole of BTH.

Similar to the aromaticity, the turbidity in this FBR-photoreactor indicated the presence of insoluble high-molecular-weight degradation products [30]. High turbidity levels further deteriorate the optical properties of the effluent and prevent light transmission to the photocatalyst. To ensure an adequate reaction rate and optimal UV light use, the turbidity should not exceed 5 NTU [36]. Figure 12d shows that the turbidity increased largely with the decrease in pH, and was appreciably higher at pH = 3.0, reaching a value of 8.5 NTU; this is in line with the most unfavourable conditions discussed above. Figure S5 compares the turbidity values with those obtained from a photocatalytic experiment performed with the GAC Hydrodarco^®^ 3000 at different pH values. At acid pH, the turbidity was much higher, approximately 10.8 NTU, thus matching the turbidity reached by the MnO_2_/GAC-3 composite, and this was most likely due to the formation of higher-molecular-weight condensation intermediates such as polyaniline, as reported by Bard and Yang [95], or macromolecules derived from BTH polymerisation [25]. In turn, at a pH of 9.0, the turbidity was 4.3 NTU and was thus lower than the threshold indicated by Bodzek and Rajca [36].

In addition, during the photocatalytic removal of ANI and BTH, the colour of the oxidised water also changed. Accordingly, these colour changes were observed by Jing et al. [19] during the oxidation of effluents containing ANI and by De Wever et al. [96] and Derco et al. [97] in effluents with BTH, with colours including pink, reddish orange, reddish brown, and light yellow. Coloured intermediates were also observed in this work during the removal of ANI and BTH, whose main intermediates could correspond to p-benzoquinone, phenol, nitrobenzene, 2-mercaptobenzothiazole and aminophenol. Thus, a higher colour intensity during the oxidation reaction represented lower pollutant mineralisation. Figure 12e shows that the colour intensity increased to extreme values at pH values of 3.0 and 7.0 (*Colour* = 1.12 AU), owing to the generation of a greater amount of oxidised chromophoric species. In turn, at pH = 9.0, both the maximum intensity (*Colour* = 0.97 AU) and the residual colour (*Colour* = 0.28 AU) were lower, owing to favourable oxidative conditions.

Dissolved oxygen was another factor that affects the photocatalytic process, given its key role during oxidation with MnO_2_/GAC-3 photocatalysts in providing efficient electron scavenging to form O_2_^•−^ and subsequently HO^•^ radicals, thus improving the efficiency of the photocatalytic process [98,99].

As shown in Figure 12f, dissolved oxygen rapidly decreased throughout the pH range, which was in agreement with the ANI and BTH oxidation rates shown in Figure 12a. The absence of oxygen at pH = 9.0 for 10 min could explain the slowdown in the mineralisation and primary degradation. Velo-Gala et al. [100] reported that in the absence of dissolved oxygen, the process became inefficient, because only the positive holes of the photocatalyst remained actively transforming water molecules into hydroxyl radicals, and the lack of an electron scavenger could not avoid the undesirable recombination phenomenon.

However, after 36 min of reaction, the oxygen concentration recovered slightly, albeit much more strongly at pH = 9.0, because the reaction system was fed from an open reservoir, which could explain this natural reoxygenation process [98].

In short, a pH of 9.0 provided the most satisfactory results in terms of oxidation, regeneration, and efficiency of the photocatalytic process, owing to its higher photocatalytic activity during the removal of ANI and BTH.

**Figure 13:**
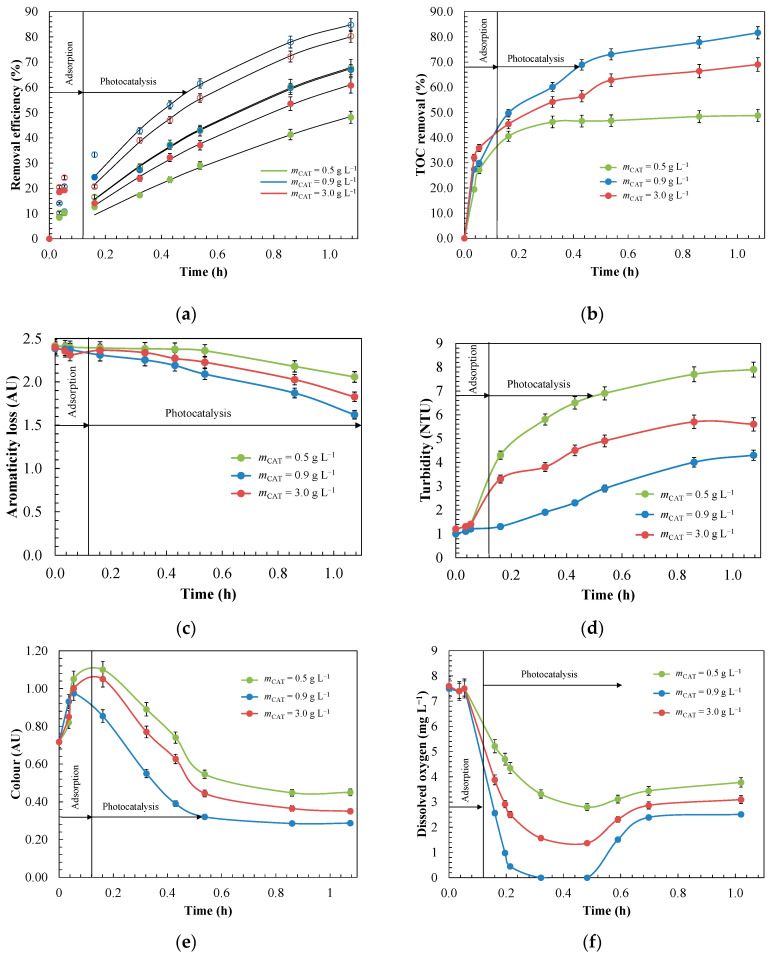
Plots showing the variation in the effect of the MnO_2_/GAC-3 catalyst dose during the photocatalytic removal of ANI and BTH from industrial effluents. Evolution of the: (**a**) primary degradation of ANI (○) and BTH (●) fitted to the apparent first-order kinetic model; (**b**) total organic carbon (TOC) removal; (**c**) aromaticity loss; (**d**) turbidity; (**e**) colour induction during aniline and benzothiazole oxidation; (**f**) dissolved oxygen. Experimental conditions: *C*_0_ = 12.0 mg L^−1^; pH = 9.0; T = 26 °C.

The dose of the catalyst used in the photocatalytic system was another key factor for guaranteeing a good performance of the system and avoiding wasting catalytic materials. The effect of the catalyst dose was assessed by varying the catalyst concentration in the 0.5–3.0 g L^−1^ range. Figure 13 showed the catalyst loading effect on ANI and BTH removal, TOC conversion profiles, colour, turbidity, aromatic ring breakage, which was expressed as absorbance at 254 nm, and dissolved oxygen.

Generally, as the catalyst dose increased, so did the number of active sites available for adsorption and degradation processes on the catalyst surface [36]. This degradation rate increased until a maximum value, after which the rate gradually decreased, with the subsequent loss of photocatalytic activity. As shown in Figure 13a,b, the best ANI and BTH removal yield was reached with a dose of 0.9 g L^−1^ and a constant of *k*_app_ of 1.75 h^−1^ and 1.04 h^−1^, respectively, with 81.6% mineralisation after 1 h of reaction was achieved. Increasing the dose favoured the generation of e^−^ and h^+^, which produced a greater amount of hydroxyl radicals, thus improving the performance of the photocatalytic process with the MnO_2_/GAC-3 catalyst [21,101]. However, upon reaching a dose of 0.9 g L^−1^, the catalytic activity worsened, with a *k*_app_ of 1.50 h^−1^ for ANI and 0.87 h^−1^ for BTH for a catalyst loading of 3.0 g L^−1^. The decrease in degradation rate might be due to reactions of free radical species with each other when generated in excess, instead of degrading ANI and BTH [21,102]. Furthermore, excessive catalyst loading could help to form agglomerates and to increase the turbidity, reducing the photocatalytic activity. As a result, the light could be scattered, thereby decreasing the radiation dose received by the photocatalyst from 155.8 W m^−2^ to 72.4 W m^−2^ with doses of 0.9 and 3.0 g L^−1^, respectively [36].

The removal yields were higher than those reported in previous studies [21] using other catalysts such as TiO_2_ in suspension in a hybrid reactor for the removal of ANI and BTH from industrial effluents. In that study, a 22-h irradiation time was required for the primary degradation of both potential contaminants. Furthermore, mineralisation only reached 50%, which may be because it was more difficult to remove the reaction intermediates than those initially present. In the present study, higher removal and mineralisation yields were achieved with shorter irradiation times than in other cases [21]. The MnO_2_/GAC-3 catalyst led to characteristics and interactions between ANI, BTH and the surface, which affected to a great extent the degradation rate, which was mostly determined by the increased generation of radical species [103].

Other parameters, such as colour, turbidity, or loss of aromaticity, which were determined by the absorbance at 254 nm (see Figure 13c–e), decreased with a dose of 0.9 g L^−1^. In addition, the lowest absorbance value at 254 nm demonstrated a high level of aromatic ring breakage, which indicated a good oxidative action under these operational conditions.

**Figure 14:**
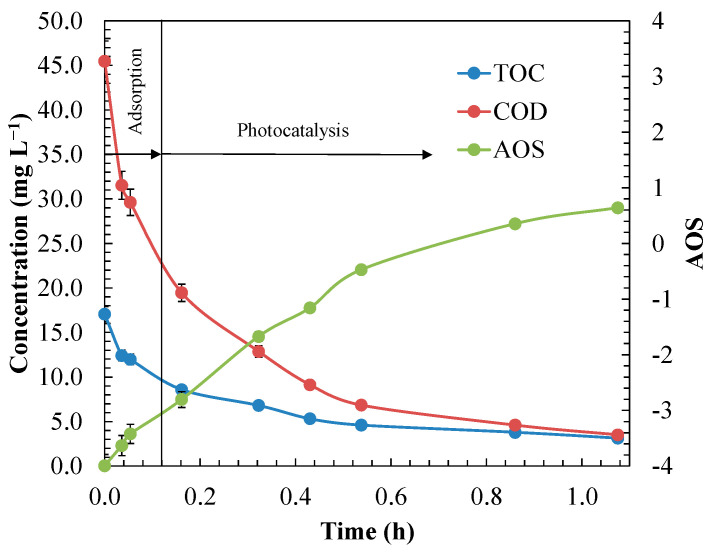
Evolution of the average oxidation state (AOS) and chemical oxygen demand (COD) during photocatalysis of ANI and BTH. Experimental conditions: *C*_0_ = 12.0 mg L^−1^; pH = 9.0; *m*_CAT_ = 0.9 g L^−1^; *Irradiation dose* = 155.8 W m^−2^; T = 26 °C.

To more practically evaluate the feasibility of the favourable operating conditions studied for the FBR-photoreactor system and its biodegradability, the average oxidation state (AOS) was estimated. This parameter is capable of indicating the degree of oxidation of complex solutions, and it is especially useful for oxidation stages at which the initial organic pollutant is a minor component compared to its degradation intermediates. In turn, the AOS indirectly provides information on biodegradability without requiring that the biological oxygen demand (BOD) be determined [104,105]. The AOS was calculated using Equation (20):

(20) $$\mathrm{AOS}=\frac{4\times \left(TOC-COD\right)}{TOC},$$

where *TOC* was the total organic carbon (mol C L^−1^), and *COD* was the chemical oxygen demand (mol O_2_ L^−1^). The parameter AOS can range from −4 at the most reduced state of C to +4 at the most oxidised state of C. AOS values from 0 to +1 would represent suitable conditions for biological organic removal [104]. Figure 14 shows the evolution of AOS over time under the best operating conditions.

As shown in Figure 14, AOS increased throughout the photocatalytic process, reaching a plateau after 1 h, with 84.7% and 67.4% primary degradation for ANI and BTH, respectively, and 81.6% mineralisation. The results indicate that a larger number of ANI and BT degradation intermediates were formed during the first 30 min of reaction, and from that time on, no relevant changes occurred in the chemical nature of the degradation intermediates, as the AOS value remained almost constant at 0.63 [106]. Consequently, the intermediates that remained in solution did not alter substantially the biodegradability of the treated effluent.

### 3.4. Stability of MnO_2_/GAC-3 after Photocatalysis and Treatment Cost Estimation

**Figure 15:**
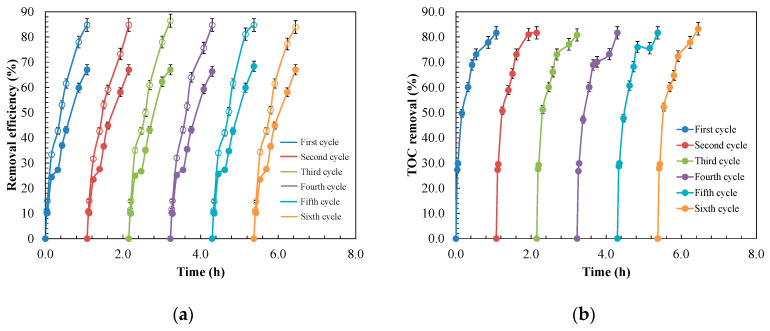
Reusability test for the photocatalytic degradation of ANI and BTH within six repeated cycles using MnO_2_/GAC-3 catalyst. Evolution of: (**a**) primary degradation of ANI (○) and BTH (●); (**b**) mineralisation. Experimental conditions: *C*_0_ = 12.0 mg L^−1^; pH = 9.0; *m*_CAT_ = 0.9 g L^−1^; *Irradiation dose* = 155.8 W m^−2^; T = 26 °C.

The ability of a photocatalyst to continuously maintain its catalytic activity for ANI and BTH removal over successive cycles is crucial for its practical application. Consequently, the MnO_2_/GAC-3 photocatalyst was subjected to six cycles of use. As shown in Figure 15, the photocatalyst showed a similar catalytic activity after six consecutive 1-h reaction cycles, maintaining an 84.7% ANI primary degradation and 67.0% BTH primary degradation, with an 81.6% mineralisation.

No changes in catalytic activity were observed. Nevertheless, Lekshemi et al. [107] observed that this could be due to a regeneration process of the MnO_2_/GAC-3 photocatalyst taking place during the reaction, according to the following mechanism:

(21) $${\mathrm{MnO}}^{\left(3+\;\mathrm{or}\;2+\right)}/\mathrm{GAC}\overset{hv}{\underset{e^{-}}{\to }}{\mathrm{MnO}}^{\left(4+\;\mathrm{or}\;3+\right)}/\mathrm{GAC}$$

(22) $$O_{2}^{-}+e^{-}\to {2O}^{-}\to O^{2-}\left({\mathrm{adsorbed}\;\mathrm{on}\;\mathrm{MnO}}_{2}/\mathrm{GAC}\right)$$

(23) $$n\left({\mathrm{Mn}}^{\left(4+\;\mathrm{or}\;3+\right)}/\mathrm{GAC}\right)+O^{2-}\left({\mathrm{adsorbed}\;\mathrm{on}\;\mathrm{MnO}}_{2}/\mathrm{GAC}\right)\to {\mathrm{MnO}}_{2}$$

However, once the catalytic properties of the photocatalyst have been spent, it must be disposed of as a solid waste. Additionally, the amount of catalyst used is small, so the volume of waste generated would be low.

**Figure 16:**
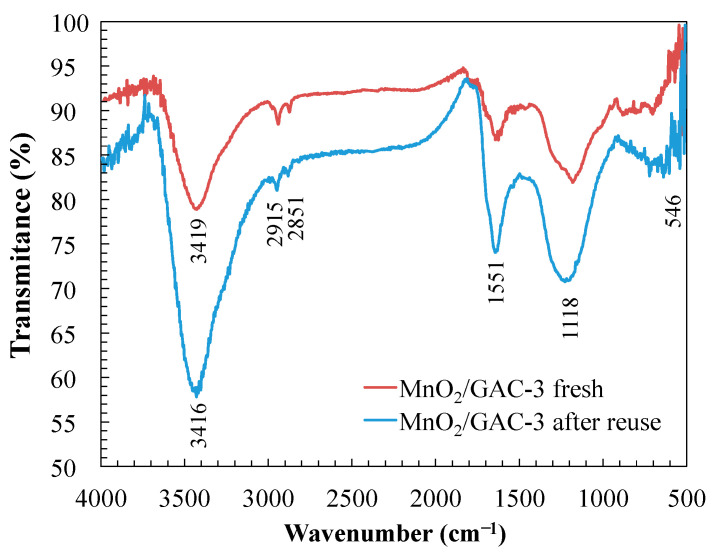
Infrared spectrum of MnO_2_/GAC-3 before and after six cycles of reuse during the photocatalytic process for ANI and BTH degradation. Experimental conditions: *C*_0_ = 12.0 mg L^−1^; pH = 9.0; *m*_CAT_ = 0.9 g L^−1^; *Irradiation dose* = 155.8 W m^−2^; T = 26 °C.

In addition, FTIR spectra of the photocatalyst were recorded before and after six reaction cycles. Infrared spectrum analysis (Figure 16) showed no new vibration bands.

However, the band at 3416 cm^−1^ associated with a bending vibration of the O–H bond qualitatively increased, owing to a higher water adsorption during the KBr pellet preparation process. The bands at 2915, 2851, and 546 cm^−1^ showed no difference from the fresh photocatalyst.

Nevertheless, the considerable increase in the absorption band corresponding to the peak at 1551 cm^−1^ could be related to the bending mode of the adsorbed water molecules when in contact with ANI [108]. Finally, the peak corresponding to the band at 1118 cm^−1^ also increased, most likely due to the adsorption and accumulation of compounds primarily formed by low-molecular-weight organic acids derived from ANI and BTH degradation [76].

The cost of an advanced oxidation process is very difficult to estimate, because factors such as mass transfer, operating conditions, and initial pollutant load can affect the degradation pathway and the oxidation kinetics, and therefore the oxidation efficiency [109]. For a direct comparison, equipment or maintenance costs were omitted, because they depend on the specific characteristics of each experimental system. Consequently, the cost of applying the FBR-photoreactor was estimated and compared to that of other systems reported in the literature that reach 90% of contaminant degradation. For this purpose, the previously determined first-order kinetic constants (*k*, h^−1^) were used to estimate the time required for 90% primary degradation of the contaminant (*t*_90_, h) according to Equation (24):

(24) $$t_{90}=\frac{2.3025851}{k}$$

Based on the *t*_90_ estimate as well as on the residence time of the reactor, the energy density (*ε*, kW L^−1^) required for treatment was calculated according to Equation (25):

(25) $$\varepsilon =\frac{E_{A}}{\mathrm{Treated}\;\mathrm{volume}},$$

where *E*_A_ (kW) is the average energy required for a treatment cycle calculated according to Equation (26):

(26) $$E_{A}=\frac{P_{\mathrm{ele}}\times t\times 1000}{V\times 60\times \log\left(\frac{C_{0}}{C_{t}}\right)},$$

where *P*_ele_ is the electrical power (kW), *t* is the irradiation time (min), *V* is the total treated volume (L), and *C*_0_ and *C*_t_ represent the ANI and BTH concentrations (mg L^−1^) at the start and at time t, respectively. The cost of a treatment cycle was calculated considering the price of electricity for industrial customers in Spain, which was 0.0882 € kWh^−1^ [110]. In Table 6, the cost of a treatment cycle is estimated and compared to others reported in the literature.

As outlined in Table 6, the FBR-photocatalytic system with the MnO_2_/GAC-3 catalyst developed in this study for the removal of ANI and BTH from an industrial effluent costs 0.17 € m^−3^ in contrast to the value of 2.19 € m^−3^ [21] for a hybrid reactor or 0.29 € m^−3^ for the FBR-Fenton system [5]. This system achieved high efficiencies, which could facilitate its industrial implementation with MnO_2_/GAC-3 catalysts.

## 4. Conclusions

A novel composite of granular activated carbon modified with MnO_2_ (MnO_2_/GAC) was prepared by hydrothermal synthesis with KMnO_4_ solutions for its use in an FBR-photoreactor for the treatment of an industrial effluent containing aniline and benzothiazole. The study of the characteristics of the material highlighted the presence of the alpha-MnO_2_ crystal in the composite by XRD analysis. Similarly, SEM, HRTEM, and AFM images showed an excellent dispersion of the MnO_2_ particles on the surface of the GAC, which was confirmed by H_2_ chemisorption (21.0%) and N_2_ adsorption, showing a 9.47% increase in the specific surface relative to the initial surface of the GAC (601.2 m^2^ g^−1^). In particular, the band gap of this composite (MnO_2_/GAC-3) was 0.95 eV, which enabled its use under visible light. XPS analysis confirmed the oxidation state (IV) of MnO_2_ deposited on GAC. The catalytic activities of three MnO_2_/GAC composites with different MnO_2_ contents were compared when applied to an industrial effluent. The MnO_2_/GAC-3 composite with 3.78% MnO_2_ content, which was determined by XRF, showed the best performance in terms of primary degradation and mineralisation. The efficiency of the process could be attributed to the use of a suitable combination of photocatalyst and FBR-photoreactor. Parameters such as geometry, particle size, surface velocity of the fluid, pH and catalyst dose were considered when establishing the most favourable conditions: pH 9.0 and a dose of 0.9 g L^−1^ for ANI and BTH removal from an industrial effluent containing 12.0 mg L^−1^, achieving 84.7% and 67.0% removal for ANI and BTH, respectively, and 81.6% mineralisation after 1 h of reaction. The degradation kinetics were modelled by fitting them to a pseudo-first-order kinetic model, thus determining the following parameters for the most favourable conditions: *k*_app, ANI_ = 1.75 h^−1^ and *k*_app, BTH_ = 1.04 h^−1^. Other parameters, such as AOS (*AOS* = 0.63), indicated that the effluent treated in this study had optimal conditions for returning to the water environment. The novel MnO_2_/GAC-3 photocatalyst showed excellent stability after six cycles, with an economic cost of treatment of 0.17 € m^−3^, thus showing a promising potential for practical applications. This study presents an efficient technology for treating industrial effluents containing organic pollutants that are not easily removed, using a novel catalytic and sustainable material.

## Figures and Tables

**Table 1:** Average physical–chemical profile of the industrial effluent containing ANI and BTH.

Parameter	Value
Aniline (mg L^−1^)	12.0 ± 0.5
Benzothiazole (mg L^−1^)	12.0 ± 0.6
Dissolved oxygen (mg O_2_ L^−1^)	7.5 ± 0.37
pH	7.8 ± 0.1
Conductivity at 20 °C (µS cm^−1^)	605 ± 30
Chemical oxygen demand (mg O_2_ L^−1^)	45.46 ± 2.27
Total organic carbon (mg C L^−1^)	16.73 ± 0.53
Temperature (°C)	26 ± 0.1
Turbidity (NTU)	1.01 ± 0.05
Aromatic ring rupture (AU)	2.453 ± 0.122
Colour (AU)	0.717 ± 0.03
Nitrite (mg NO_2_ L^−1^)	0.041 ± 0.001
Nitrate (mg NO_3_ L^−1^)	1.6 ± 0.1
Chloride (mg Cl L^−1^)	31.7 ± 1.6
Total phosphorus (mg P L^−1^)	0.05 ± 0.01
Phosphates (mg PO_4_ L^−1^)	0.07 ± 0.01
Total ammonia (mg NH_4_ L^−1^)	0.14 ± 0.01

**Table 2:** Results from N_2_ physisorption of the synthesised α-MnO_2_ nanoparticles, commercial active carbon Hydrodarco^®^ 3000, and three MnO_2_/GAC composites prepared in this study.

Sample	S_BET_, m^2^ g^−1^	S_ext_, m^2^ g^−1^	V_T_, cm^3^ g^−1^	Vμ, cm^3^ g^−1^	V_M_, cm^3^ g^−1^	V_M_/V_T_·100 %	V_μ_/V_T_·100 %	D_P_, Å
α-MnO_2_	23.5	10.5	0.07	0.05	0.02	28.6	71.4	127.7
Hydrodarco^®^ 3000 GAC	601.2	276.4	0.51	0.14	0.35	68.6	27.5	83.98
MnO_2_/GAC-1	496.1	294.1	0.49	0.09	0.38	77.6	18.0	81.28
MnO_2_/GAC-2	556.3	305.8	0.51	0.1	0.38	74.5	19.6	80.50
MnO_2_/GAC-3	664.1	345.49	0.62	0.13	0.48	77.4	21.0	88.03

**Table 3:** Analysis and chemical properties of the synthesised α-MnO_2_ nanoparticles, commercial activated carbon Hydrodarco^®^ 3000, and the three MnO_2_/GAC composites prepared in this study.

Properties	Sample				
	α-MnO_2_	Hydrodarco^®^ 3000 GAC	MnO_2_/GAC-1	MnO_2_/GAC-2	MnO_2_/GAC-3
pH_PZC_	2.60	7.25	7.10	6.85	6.61
*Disp*, %	—	—	15.5	17.3	21.0
MnO_2_, %	N/A	N/A	1.34	2.07	3.78
Al_2_O_3_, %	—	0.66	0.41	0.57	0.77
Fe_2_O_3_, %	—	0.26	0.15	0.36	0.34
SiO_2_, %	—	6.96	7.27	7.72	6.02
MgO, %	—	0.15	0.23	0.06	0.23
CaO, %	—	0.11	0.15	0.11	0.09
Na_2_O, %	—	0.01	0.01	0.01	0.01
K_2_O, %	—	0.21	0.18	0.30	0.16
TiO_2_, %	—	0.17	0.14	0.38	0.10
P_2_O_5_, %	—	0.01	0.01	0.01	0.01
S, %	—	0.21	0.34	0.22	0.12

**Table 4:** Results from the kinetic modelling of the photocatalysis and adsorption process during ANI and BTH removal using the photocatalyst MnO_2_/GAC-3. Experimental conditions: *C*_0_ = 12.0 mg L^−1^; pH = 9.0; *m*_CAT_ = 0.9 g L^−1^; *Irradiation dose* = 155.8 W m^−2^.

Kinetic Model	Parameters	Process			
		Adsorption		Photocatalysis	
		Aniline	Benzothiazole	Aniline	Benzothiazole
Pseudo-1st-order	*q*_e_ (mg g^−1^)	18.16	14.12	1.81	1.84
	*k*_1_ (h^−1^)	0.479	0.509	1.208	0.914
	*R* ^2^	0.663	0.688	0.996	0.988
Pseudo-2nd-order	*q*_e_ (mg g^−1^)	45.25	30.03	2.14	1.90
	*k*_2_ (g mg^−1^ h^−1^)	0.084	0.113	1.442	1.169
	*R* ^2^	0.995	0.988	0.950	0.844

**Table 5:** Summary of the kinetic parameters and coefficients of determination (*R*^2^) of the photocatalytic process using MnO_2_/GAC-3 composites after reaction for 1 h.

Effect	*k*_app, ANI_ (h^−1^)	*t*_1/2, ANI_ (h)	*R* ^2^	*k*_app, BTH_ (h^−1^)	*t*_1/2, BTH_ (h)	*R* ^2^	*Irradiation Dose* (W m^−2^)
**pH** ^1^							
3.0	0.87	0.79	0.98	0.52	1.33	0.99	155.8
7.0	1.31	0.53	0.99	0.77	0.90	0.99	155.8
9.0	1.75	0.39	0.99	1.04	0.66	0.99	155.8
**MnO_2_/GAC-3** **dosage** ^2^ **(g L^−1^)**							
0.5	1.05	0.66	0.99	0.61	1.12	0.98	199.6
0.9	1.75	0.39	0.99	1.04	0.66	0.99	155.8
3.0	1.50	0.46	0.99	0.87	0.79	0.99	72.4

^1^ Experiments performed with a catalyst mass of 0.9 g L^−1^. ^2^ Assessment of the catalyst loading effect at pH = 9.0.

**Table 6:** Procedure and comparison of ANI and BTH treatment cost estimates.

Process	Pollutant	*k*	Treated Volume (L)	*P*_ele_ (kW)	*t*_90_ (h)	*ε* (kW L^−1^)	*V* (L)	*V* × *ε* (kW)	Cost (€ m^−3^)	Ref.
FBR-Fenton	ANI	1.739 h^−1^	0.859	0.012	1.32	0.014	238.26	3.33	0.29	[5]
Ozone	ANI	2.003 h^−1^	1.0	0.200	1.14	235.43	1.0	235.43	0.81	[19]
Ozone	BTH	1.296 h^−1^	5.0	0.200	1.77	1.84	5.0	9.20	2.76	[9]
Photocatalysis	ANI and BTH mixture	0.341 h^−1^ (ANI) 0.091 h^−1^ (BTH)	2.15	0.026	6.75 (ANI) 25.30 (BTH)	4.97	16.0	79.59	2.19	[21]
FBR-Photocatalytic	ANI and BTH mixture	1.75 h^−1^ (ANI) 1.04 h^−1^ (BTH)	1.63	0.025	1.31 (ANI) 2.21 (BTH)	0.057	10.0	0.57	0.17 ^1^	This work

^1^ Considering the electric energy consumption of pumping.

## Supplementary Materials

The following are available online at https://www.mdpi.com/article/10.3390/ma14185207/s1, Figure S1: Spectrum of the UV-A lamp used in the experimental equipment, Figure S2: EDX spectra of the materials used in the study, Figure S3: Determination of the H_2_ monolayer chemisorbed on the surface of the three MnO_2_/GAC composites synthesised in this study, Figure S4: pH effect on the efficiency of the ANI and BTH removal process using GAC Hydrodarco^®^ 3000 during the adsorption and photocatalysis processes, Figure S5: pH effect on turbidity during the photocatalysis of industrial effluents containing ANI and BTH using the GAC Hydrodarco^®^ 3000, Table S1: Previous studies of photocatalysis using manganese oxides supported on carbonaceous materials, Table S2: Limit of quantification values (LOQ) and limit of detection values (LOD) of different parameters, Table S3: Linearity values of different parameters, Table S4: Specificity values of different parameters, Table S5: Accuracy values of different parameters, Table S6: Precision values of different parameters.
