# Supplementary material for: Removal of Aniline and Benzothiazole Wastewaters Using an Efficient MnO2/GAC Catalyst in a Photocatalytic Fluidised Bed Reactor
Source: Materials (Basel). 2021 Sep 10;14(18):5207. doi: 10.3390/ma14185207 (PMC8467099; doi:10.3390/ma14185207)
Supplement: Supplementary file 1 [file materials-14-05207-s001.zip › materials-1365356-supplementary.pdf]

materials

# Supplementary Material

# Removal of Aniline and Benzothiazole Wastewaters Using an Efficient $MnO_2$/GAC Catalyst in a Photocatalytic Fluidised Bed Reactor

**Cristian Ferreiro [1,*], Natalia Villota [2], José Ignacio Lombraña [1], María J. Rivero [3], Verónica Zúñiga [4] and José Miguel Rituerto [4]**

[1] Department of Chemical Engineering, Faculty of Science and Technology, University of the Basque Country UPV/EHU, Barrio Sarriena s/n, 48940 Leioa, Spain; ji.lombrana@ehu.eus
[2] Department of Chemical and Environmental Engineering, Faculty of Engineering Vitoria-Gasteiz, University of the Basque Country UPV/EHU, Nieves Cano 12, 01006 Vitoria-Gasteiz, Spain; natalia.villota@ehu.eus
[3] Department of Chemical and Biomolecular Engineering, University of Cantabria, 39005 Santander, Spain; riveromj@unican.es
[4] General Química, S.A.U. (Grupo Dynasol), 01213 Lantaron, Spain; veronica.zuniga@repsol.com (V.Z.); jrituertol@repsol.com (J.M.R.)
* Correspondence: cristian.ferreiro@ehu.eus; Tel.: +34-946-012-512

**Table S1.** Previous studies of photocatalysis using manganese oxides supported on carbonaceous materials.

| Catalyst | Operating conditions | Comments | Ref. |
|---|---|---|---|
| $\alpha$-$MnO_2$ | *[Cat.]* = 0.025 g<br>$\tau$= 1.655 × $10^{-3}$ mol $h^{-1}$ g $cat^{-1}$<br>*T* = 25 °C<br>*Time* = 8 h<br>*[% removal]* = 100 | Up to 100% conversion into acetone using an amorph $MnO_2$ photocatalytic system for 2-propanol removal. The $\alpha$-$MnO_2$ phase was more active and regenerable than other manganese oxides. | [1] |
| $\alpha$-$MnO_2$/CNT | *[Cat.]* = 0.4 g $L^{-1}$<br>$C_0$ = 20 mg $L^{-1}$<br>*T* = 25 °C<br>*Time* = 75 min<br>*[% removal]* = 24 | Compared different crystalline phases of $MnO_2$. Found that $\alpha$-$MnO_2$ particles supported on carbon nanotubes (CNT) improved the surface area of the hybrid structure and had a better performance under visible light. | [2] |
| $MnO_2$/AC | *[Cat.]* = 3.0 g $L^{-1}$<br>$C_0$ = 60 mg $L^{-1}$<br>*T* = 25 °C<br>*Time* = 5 min<br>*[% removal]* = 98.53 | Compared the catalytic activity of $MnO_2$ nanoparticles to that of $MnO_2$/AC composites. The composite removed 31.96% more Congo Red (CR) dye than the nanoparticles. The presence of mineral ions led to a higher dye degradation rate. | [3] |
| GO/$MnO_2$ | *[Cat.]* = 0.5 g $L^{-1}$<br>$C_0$ = 60 mg $L^{-1}$<br>*T* = 25 °C<br>*Time* = 120 min<br>*[% removal]* = 98 | Observed that the catalytic activity during Reactive Black 5 (RB5) removal was higher than that of their starting materials. The presence of graphene oxide with a 20% $MnO_2$ content helped to increase the generation of hydroxyl radicals and facilitated their separation from the solution, in contrast to $MnO_2$ nanoparticles. | [4] |
| $MnO_2$/AC | *[Cat.]* = 0.5 g $L^{-1}$<br>$C_0$ = 100 mg $L^{-1}$<br>*T* = 25 °C<br>*Time* = 60 min<br>*[% removal]* = 85 | Impregnated an activated carbon with a 20% B$MnO_2$ content. Concluded that carbon impregnated with B$MnO_2$ showed a higher potential for RB5 degradation. | [5] |

**Photocatalytic reaction mechanism with $MnO_2$/GAC composites**

The improvement can be attributed to the reaction mechanisms which occur on the surface of the catalyst. The trigger mechanism begins with light absorption ($h\upsilon$), generating electron-hole ($e^-$ - $h^+$) pairs, which act as reducing and oxidising agents (Eq. 1). During oxidation, water (Eq. 2) or hydroxide ions ($OH^-$) (Eq. 3) adsorbed on the catalyst generate hydroxyl radicals ($HO^\bullet$), while oxygen prevents the recombination of electron-hole pairs (Eq. 4). Subsequently, when irradiating a photon of energy equal to or greater than the band gap energy, the excitation of electrons in the conduction band (CB) is produced and it generates an equal number of holes in the valence band (VB) (Eqs. 5– 7). At that point, Eqs. 8–9, the high oxidative species generated are responsible for ANI and BTH oxidation to other degradation by-products.

According to Ameta [6] and Rahmat et al. [7], the equations below describe a plausible mechanism of photocatalysis of an activated carbon material modified with $MnO_2$ ($MnO_2$/GAC):

$$MnO_2/GAC \xrightarrow{h\upsilon} MnO_2/GAC\left(e^- + h^+\right) \quad (1)$$

$$MnO_2/GAC + h^+ + H_2O \rightarrow MnO_2/GAC + H^+ + HO^\bullet \quad (2)$$

$$MnO_2/GAC + h^+ + OH^- \rightarrow MnO_2/GAC + HO^\bullet \quad (3)$$

$$MnO_2/GAC + e^- + O_2 \rightarrow MnO_2/GAC + O_2^{-\bullet} \quad (4)$$

$$O_2^{-\bullet} + H^+ \rightarrow HO_2^\bullet \quad (5)$$

$$HO_2^\bullet + HO_2^\bullet \rightarrow H_2O_2 + O_2 \quad (6)$$

$$H_2O_2 \xrightarrow{h\upsilon} HO^\bullet \quad (7)$$

$$HO^\bullet + ANI + BTH \rightarrow \text{Oxidation products} \quad (8)$$

$$HO_2^\bullet + ANI + BTH \rightarrow \text{Oxidation products} \quad (9)$$

Achieving a high removal efficiency during the photocatalytic process requires [8–10]: i) an adequate diffusion of contaminants from the liquid phase to the surface of the $MnO_2$ catalyst, ii) a strong adsorption of organic pollutants on the surface of $MnO_2$, iii) redox reactions at the catalyst surface level, and iv) desorption of oxidation products. Based on these conditions, the reactor could play a key role not only in terms of degradation but also in terms of ANI and BTH mineralisation. Therefore, the application of advanced oxidation processes (AOPs) with suitable reactor technology can reduce catalyst damage whilst increasing reuse [11].

**Analytical methods for influent characterization**

Nitrite, nitrates, chlorides and phosphates were measured by ion chromatography (IC) using a Dionex Model 2010i Ion Chromatograh (Dionex, Sunnyvale, CA, USA) with a conductivity detector, Model CDM-1 (Dionex, Sunnyvale, CA, USA). Chromatographic separation was achieved using a IonPac-AS4 (250 × 4.0 mm, Dionex, Sunnyvale, CA, USA) column with a IonPac-AG4 guard column (50 × 4.0 mm, Dionex, Sunnyvale, CA, USA). The eluent used consisted in a mixture of 3.5 mM $NaHCO_3$ and 2.0 mM $Na_2CO_3$. A sample volume of 50 μL was injected according with the method described by Urasa and Ferede [12].

**Quality assurance (QA) and quality control (QC) protocols**

The protocols followed for the determination of the different parameters listed in Table 1 were described below. The samples were subjected to drastic conditions to acid hydrolysis (1N HCl), basic hydrolysis (1N NaOH), sunlight and temperature (30 °C). Subsequently, the amount recovered was determined in triplicate after 7 days.

**Table S2.** Limit of quantification values (LOQ) and limit of detection values (LOD) of different parameters.

| Parameter | LOQ | LOD |
|---|---|---|
| Aniline (mg $L^{-1}$) | 0.01 | 30.0 |
| Benzothiazole (mg $L^{-1}$) | 0.02 | 24.0 |
| Nitrite (mg $NO_2$ $L^{-1}$) | 0.0010 | 50.0 |
| Nitrate (mg $NO_3$ $L^{-1}$) | 0.0015 | 75.0 |
| Chloride (mg Cl $L^{-1}$) | 0.003 | 80.0 |
| Total phosphorus (mg P $L^{-1}$) | 0.002 | 32.0 |
| Phosphates (mg $PO_4$ $L^{-1}$) | 0.001 | 46.0 |

**Table S3.** Linearity values of different parameters.

| Parameter | Range | Regression equation | $R^2$ |
|---|---|---|---|
| Aniline (mg $L^{-1}$) | 0.1-20.0 | y = 3.780 x + 2.350 | 0.998 |
| Benzothiazole (mg $L^{-1}$) | 0.1-20.0 | y = 7.033 x + 8.196 | 0.998 |
| Nitrite (mg $NO_2$ $L^{-1}$) | 0.002-0.1 | y = 5.280 x + 3.040 | 0.993 |
| Nitrate (mg $NO_3$ $L^{-1}$) | 0.002-4.0 | y = 2.098 x + 9.636 | 0.998 |
| Chloride (mg Cl $L^{-1}$) | 0.01-65.0 | y = 2.558 x + 3.146 | 0.991 |
| Total phosphorus (mg P $L^{-1}$) | 0.01-0.1 | y = 3.973 x + 5.480 | 0.995 |
| Phosphates (mg $PO_4$ $L^{-1}$) | 0.01-0.3 | y = 3.108 x + 2.721 | 0.998 |

**Table S4.** Specificity values of different parameters.

| Parameter | Condition | Amount added | Amount recovered | Degradation (%) |
|---|---|---|---|---|
| Aniline (mg $L^{-1}$) | Acidic degradation | 12.0 | 11.7 | 1.87 |
| | Alkaline degradation | 12.0 | 11.7 | 2.12 |
| | Solar light | 12.0 | 11.6 | 3.04 |
| Benzothiazole (mg $L^{-1}$) | Acidic degradation | 12.0 | 11.8 | 1.15 |
| | Alkaline degradation | 12.0 | 11.9 | 0.71 |
| | Solar light | 12.0 | 11.8 | 0.98 |
| Nitrite (mg $NO_2$ $L^{-1}$) | Acidic degradation | 0.050 | 0.049 | 1.54 |
| | Alkaline degradation | 0.050 | 0.048 | 2.2 |
| | Solar light | 0.050 | 0.048 | 2.03 |
| Nitrate (mg $NO_3$ $L^{-1}$) | Acidic degradation | 1.50 | 1.47 | 1.64 |
| | Alkaline degradation | 1.50 | 1.44 | 3.72 |
| | Solar light | 1.50 | 1.48 | 0.99 |
| Chloride (mg Cl $L^{-1}$) | Acidic degradation | 30.0 | 29.3 | 2.31 |
| | Alkaline degradation | 30.0 | 28.9 | 3.47 |
| | Solar light | 30.0 | 29.6 | 1.02 |
| Total phosphorus (mg P $L^{-1}$) | Acidic degradation | 0.05 | 0.04 | 0.8 |
| | Alkaline degradation | 0.05 | 0.04 | 1.74 |
| | Solar light | 0.05 | 0.05 | 2.8 |
| Phosphates (mg $PO_4$ $L^{-1}$) | Acidic degradation | 0.100 | 0.097 | 2.7 |
| | Alkaline degradation | 0.100 | 0.099 | 0.85 |
| | Solar light | 0.100 | 0.096 | 3.87 |

**Table S5.** Accuracy values of different parameters.

| Parameter | Range | Recovery (Mean ± % RSD) |
|---|---|---|
| Aniline (mg $L^{-1}$) | 0.1-20.0 | 100.91 ± 0.024 |
| Benzothiazole (mg $L^{-1}$) | 0.1-20.0 | 100.24 ± 0.030 |
| Nitrite (mg $NO_2$ $L^{-1}$) | 0.002-0.1 | 100.14 ± 0.020 |
| Nitrate (mg $NO_3$ $L^{-1}$) | 0.002-4.0 | 100.08 ± 0.007 |
| Chloride (mg Cl $L^{-1}$) | 0.01-65.0 | 100.05 ± 0.026 |
| Total phosphorus (mg P $L^{-1}$) | 0.01-0.1 | 100.32 ± 0.013 |
| Phosphates (mg $PO_4$ $L^{-1}$) | 0.01-0.3 | 100.56 ± 0.011 |

**Table S6.** Precision values of different parameters.

| Parameter | Concentration | Standard solution | | Sample solution | | Mean | SD | % RSD |
|---|---|---|---|---|---|---|---|---|
| | | Intra-day precision | Inter-day precision | Intra-day precision | Inter-day precision | | | |
| Aniline (mg $L^{-1}$) | 12.0 | 12.020 | 12.013 | 12.051 | 12.042 | 12.0315 | 0.0179 | 0.149 |
| Benzothiazole (mg $L^{-1}$) | 12.0 | 12.090 | 12.047 | 12.100 | 12.084 | 12.08025 | 0.0231 | 0.191 |
| Nitrite (mg $NO_2$ $L^{-1}$) | 0.05 | 0.0510 | 0.0490 | 0.0521 | 0.0514 | 0.050875 | 0.0013 | 2.614 |
| Nitrate (mg $NO_3$ $L^{-1}$) | 1.50 | 1.5039 | 1.5010 | 1.5120 | 1.5109 | 1.50695 | 0.0053 | 0.354 |
| Chloride (mg Cl $L^{-1}$) | 30.0 | 30.017 | 30.011 | 30.029 | 30.024 | 30.02025 | 0.0078 | 0.026 |
| Total phosphorus (mg P $L^{-1}$) | 0.05 | 0.059 | 0.053 | 0.051 | 0.055 | 0.0545 | 0.0034 | 6.267 |
| Phosphates (mg $PO_4$ $L^{-1}$) | 0.10 | 0.1024 | 0.1031 | 0.1015 | 0.1043 | 0.102825 | 0.0011 | 1.148 |

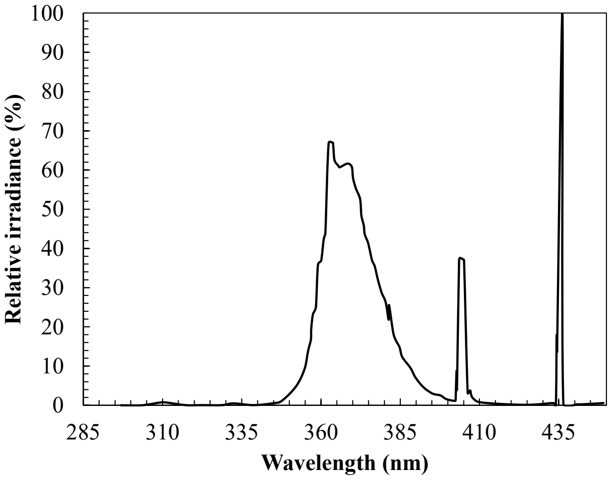


**Figure S1.** Spectrum of the UV-A lamp used in the experimental equipment.

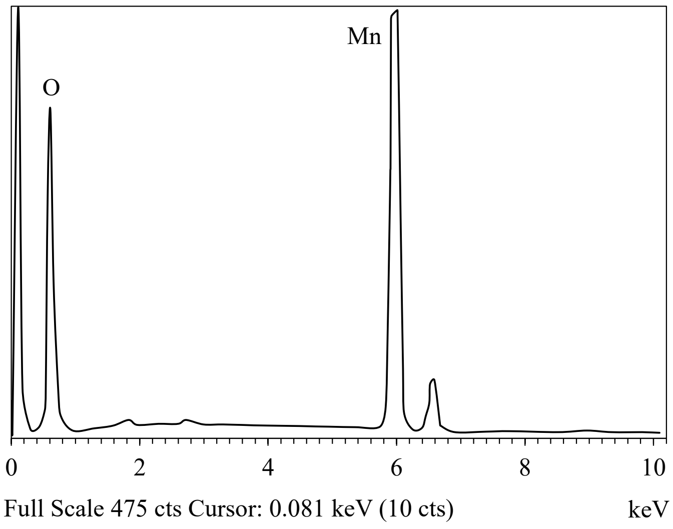


(**a**)

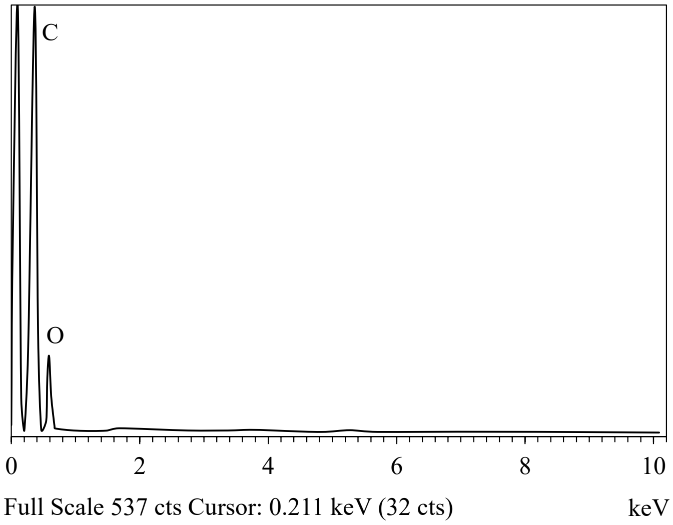


(**b**)

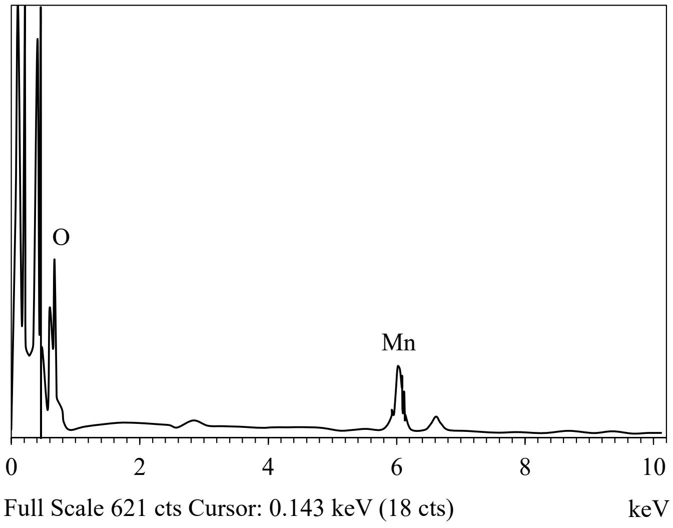


(**c**)

**Figure S2.** EDX spectra of the materials used in the study: (**a**) synthesised $MnO_2$ powder; (**b**) Commercial activated carbon Hydrodarco® 3000; (**c**) Synthesized $MnO_2$/GAC-3 composite.

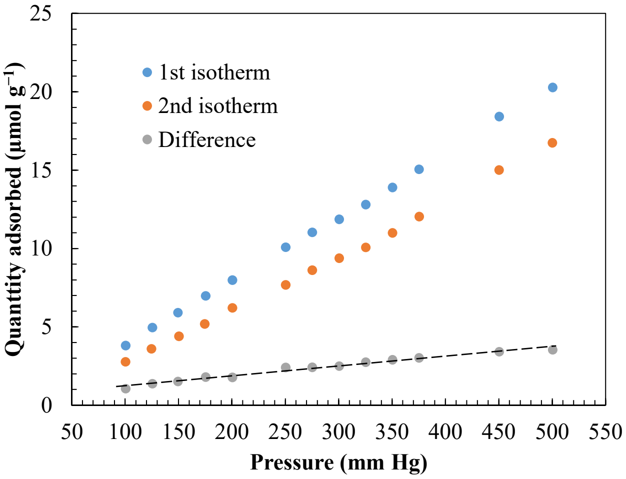


(**a**)

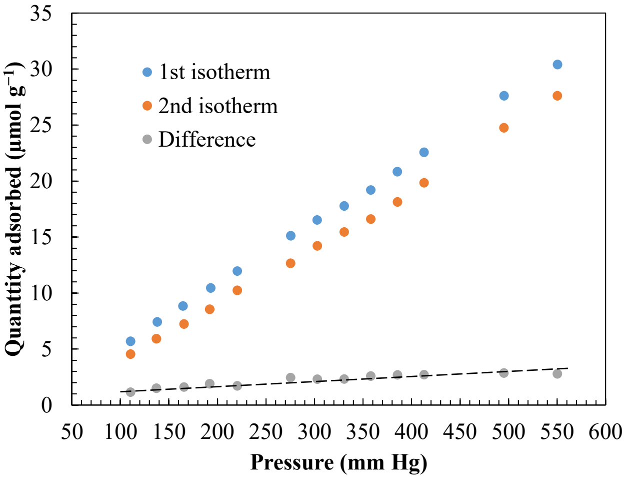


(**b**)

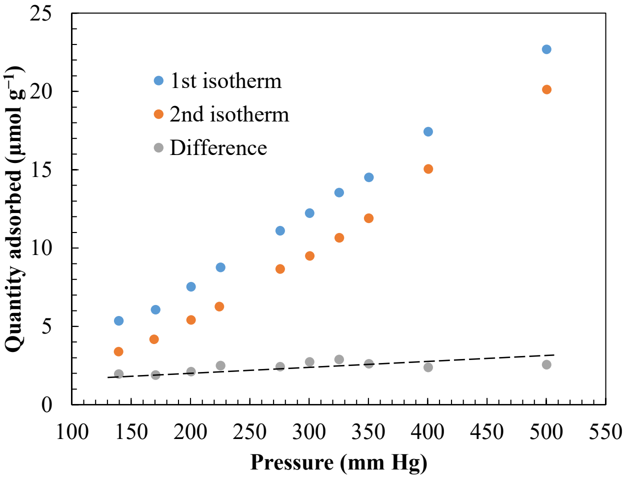


(**c**)

**Figure S3.** Determination of the $H_2$ monolayer chemisorbed on the surface of the three $MnO_2$/GAC composites synthesised in this study: (**a**) $MnO_2$/GAC-1; (**b**) $MnO_2$/GAC-2; (**c**) $MnO_2$/GAC-3.

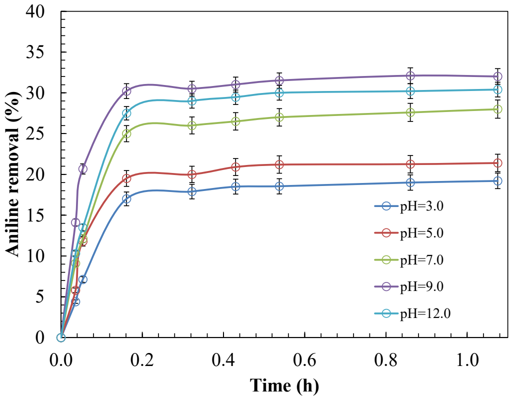


(**a**)

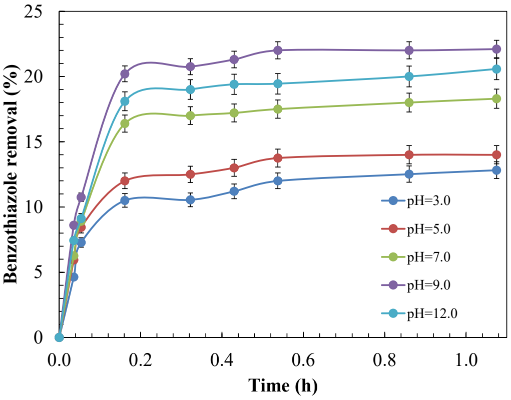


(**b**)

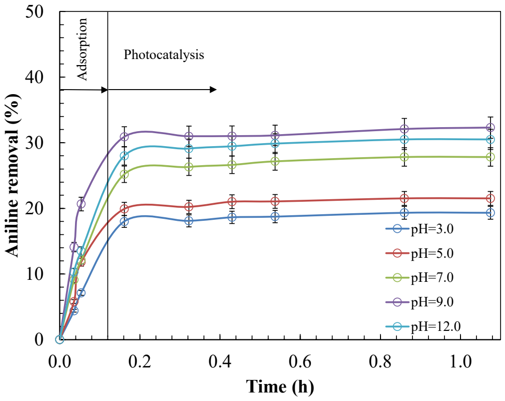


(**c**)

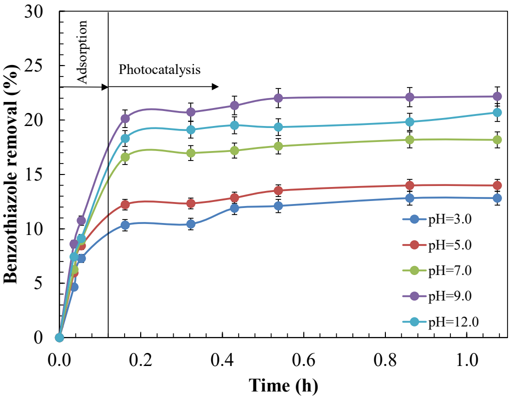


(**d**)

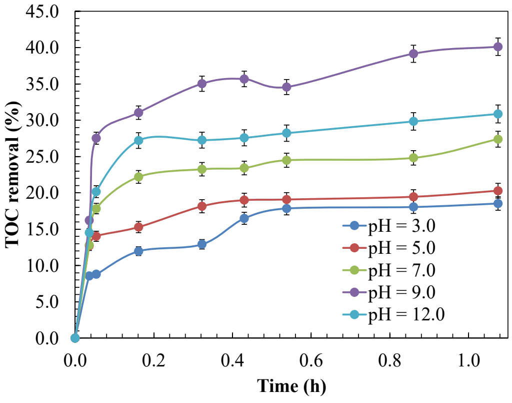


(**e**)

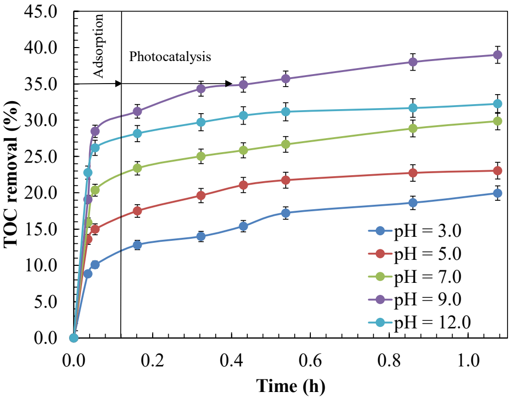


(**f**)

**Figure S4.** pH effect on the efficiency of the ANI and BTH removal process using GAC Hydrodarco® 3000 during the adsorption and photocatalysis processes. Evolution of the: (**a**,**b**) Adsorption; (**c**,**d**) Photocatalysis in primary degradation (**e**,**f**) and in mineralisation in the adsorption and photocatalysis processes, respectively. Experimental conditions: $C_0$ = 12.0 mg $L^{-1}$; $m_{CAT}$ = 0.9 g $L^{-1}$; *Irradiation dose* = 155.8 W $m^{-2}$; T = 26 °C.

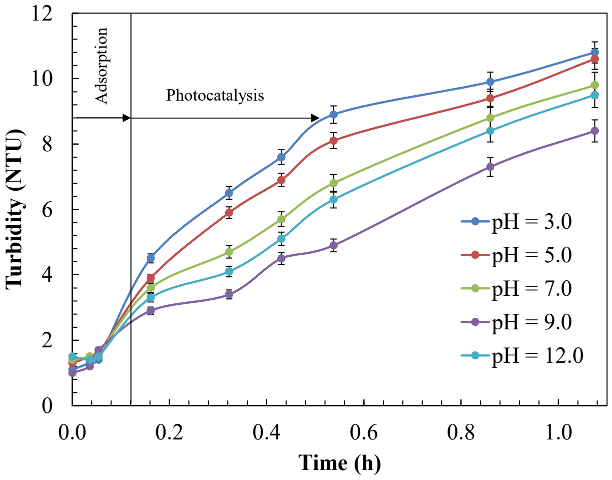


**Figure S5.** pH effect on turbidity during the photocatalysis of industrial effluents containing ANI and BTH using the GAC Hydrodarco® 3000. Experimental conditions: $C_0$ = 12.0 mg L$^{-1}$; $m_{CAT}$ = 0.9 g L$^{-1}$; *Irradiation dose* = 155.8 W m$^{-2}$; T = 26 °C.

## References

1. Cao, H.; Suib, S.L. Highly Efficient Heterogeneous Photooxidation of 2-Propanol to Acetone with Amorphous Manganese Oxide Catalysts. *J. Am. Chem. Soc.* **1994**, *116*, 5334–5342, doi:10.1021/ja00091a044.
2. Warsi, M.F.; Bilal, M.; Zulfiqar, S.; Khalid, M.U.; Agboola, P.O.; Shakir, I. Enhanced Visible Light Driven Photocatalytic Activity of $MnO_2$ Nanomaterials and Their Hybrid Structure with Carbon Nanotubes. *Mater. Res. Express* **2020**, *7*, 105015, doi:10.1088/2053-1591/abbf8d.
3. Khan, I.; Sadiq, M.; Khan, I.; Saeed, K. Manganese Dioxide Nanoparticles/Activated Carbon Composite as Efficient UV and Visible-Light Photocatalyst. *Environ. Sci. Pollut. Res.* **2019**, *26*, 5140–5154, doi:10.1007/s11356-018-4055-y.
4. Saroyan, H.; Kyzas, G.Z.; Deliyanni, E.A. Effective Dye Degradation by Graphene Oxide Supported Manganese Oxide. *Processes* **2019**, *7*, 40, doi:10.3390/pr7010040.
5. Saroyan, H.S.; Arampatzidou, A.; Voutsa, D.; Lazaridis, N.K.; Deliyanni, E.A. Activated Carbon Supported $MnO_2$ for Catalytic Degradation of Reactive Black 5. *Colloids Surf. A-Physicochem. Eng. Asp.* **2019**, *566*, 166–175, doi:10.1016/j.colsurfa.2019.01.025.
6. Ameta, S. *Advanced Oxidation Processes for Wastewater Treatment: Emerging Green Chemical Technology*; Academic Press, San Diego, CA, USA, **2018**; ISBN 978-0-12-810499-6.
7. Rahmat, M.; Rehman, A.; Rahmat, S.; Bhatti, H.N.; Iqbal, M.; Khan, W.S.; Bajwa, S.Z.; Rahmat, R.; Nazir, A. Highly Efficient Removal of Crystal Violet Dye from Water by $MnO_2$ Based Nanofibrous Mesh/Photocatalytic Process. *J. Mater. Res. Technol.* **2019**, *8*, 5149–5159, doi:10.1016/j.jmrt.2019.08.038.
8. Tang, N.; Tian, X.; Yang, C.; Pi, Z.; Han, Q. Facile Synthesis of $\alpha$-$MnO_2$ Nanorods for High-Performance Alkaline Batteries. *J. Phys. Chem. Solids* **2010**, *71*, 258–262, doi:10.1016/j.jpcs.2009.11.016.
9. Lin, H.; Chen, D.; Liu, H.; Zou, X.; Chen, T. Effect of $MnO_2$ Crystalline Structure on the Catalytic Oxidation of Formaldehyde. *Aerosol Air Qual. Res.* **2017**, *17*, 1011–1020, doi:10.4209/aaqr.2017.01.0013.
10. Wang, B.; Zhang, H.; Wang, F.; Xiong, X.; Tian, K.; Sun, Y.; Yu, T. Application of Heterogeneous Catalytic Ozonation for Refractory Organics in Wastewater. *Catalysts* **2019**, *9*, 241, doi:10.3390/catal9030241.
11. Farines, V.; Baig, S.; Albet, J.; Molinier, J.; Legay, C. Ozone Transfer from Gas to Water in a Co-Current Upflow Packed Bed Reactor Containing Silica Gel. *Chem. Eng. J.* **2003**, *91*, 67–73, doi:10.1016/S1385-8947(02)00137-7.
12. Urasa, I.T.; Ferede, F. The Determination of Phosphates Using Ion Chromatography: An Evaluation of Influential Factors. *Int. J. Environ. Anal. Chem.* **1986**, *23*, 189–206, doi:10.1080/03067318608076445.
